# Distinct Dictation of Japanese Encephalitis Virus-Induced Neuroinflammation and Lethality via Triggering TLR3 and TLR4 Signal Pathways

**DOI:** 10.1371/journal.ppat.1004319

**Published:** 2014-09-04

**Authors:** Young Woo Han, Jin Young Choi, Erdenebelig Uyangaa, Seong Bum Kim, Jin Hyoung Kim, Bum Seok Kim, Koanhoi Kim, Seong Kug Eo

**Affiliations:** 1 College of Veterinary Medicine and Bio-Safety Research Institute, College of Natural Science, Chonbuk National University, Jeonju, Republic of Korea; 2 Department of Biology, College of Natural Science, Chonbuk National University, Jeonju, Republic of Korea; 3 Department of Pharmacology, School of Medicine, Pusan National University, Yangsan, Republic of Korea; Oregon Health and Science University, United States of America

## Abstract

Japanese encephalitis (JE) is major emerging neurologic disease caused by JE virus. To date, the impact of TLR molecules on JE progression has not been addressed. Here, we determined whether each TLR modulates JE, using several TLR-deficient mouse strains (TLR2, TLR3, TLR4, TLR7, TLR9). Surprisingly, among the tested TLR-deficient mice there were contrasting results in TLR3^−/−^ and TLR4^−/−^ mice, *i.e.* TLR3^−/−^ mice were highly susceptible to JE, whereas TLR4^−/−^ mice showed enhanced resistance to JE. TLR3 ablation induced severe CNS inflammation characterized by early infiltration of inflammatory CD11b^+^Ly-6C^high^ monocytes along with profoundly increased viral burden, proinflammatory cytokine/chemokine expression as well as BBB permeability. In contrast, TLR4^−/−^ mice showed mild CNS inflammation manifested by reduced viral burden, leukocyte infiltration and proinflammatory cytokine expression. Interestingly, TLR4 ablation provided potent *in vivo* systemic type I IFN innate response, as well as *ex vivo* type I IFN production associated with strong induction of antiviral PRRs (RIG-I, MDA5), transcription factors (IRF-3, IRF-7), and IFN-dependent (PKR, Oas1, Mx) and independent ISGs (ISG49, ISG54, ISG56) by alternative activation of IRF3 and NF-κB in myeloid-derived DCs and macrophages, as compared to TLR3^−/−^ myeloid-derived cells which were more permissive to viral replication through impaired type I IFN innate response. TLR4 ablation also appeared to mount an enhanced type I IFN innate and humoral, CD4^+^ and CD8^+^ T cell responses, which were mediated by altered immune cell populations (increased number of plasmacytoid DCs and NK cells, reduced CD11b^+^Ly-6C^high^ monocytes) and CD4^+^Foxp3^+^ Treg number in lymphoid tissue. Thus, potent type I IFN innate and adaptive immune responses in the absence of TLR4 were closely coupled with reduced JE lethality. Collectively, these results suggest that a balanced triggering of TLR signal array by viral components during JE progression could be responsible for determining disease outcome through regulating negative and positive factors.

## Author Summary

Japanese encephalitis (JE) is major emerging encephalitis, and more than 60% of global population inhabits JE endemic areas. The etiological virus is currently spreading to previously unaffected regions due to rapid changes in climate and demography. However, the impact of TLR molecules on JE progression has not been addressed to date. We found that the distinct outcomes of JE progression occurred in TLR3 and TLR4-dependent manner, *i.e.* TLR3^−/−^ mice were highly susceptible, whereas TLR4^−/−^ mice showed enhanced resistance to JE. TLR3 ablation induced severe CNS inflammation manifested by early CD11b^+^Ly-6C^high^ monocyte infiltration, high expression of proinflammatory cytokines, as well as increased BBB permeability. In contrast, TLR4 ablation provided potent type I IFN innate response in infected mice, as well as in myeloid-derived cells closely associated with strong induction of antiviral ISG genes, and also resulted in enhanced humoral, CD4+, and CD8+ T cell responses along with altered plasmacytoid DC and CD4^+^Foxp3^+^ Treg number. Thus, potent type I IFN innate and adaptive immune responses in the absence of TLR4 were coupled with reduced JE lethality. Our studies provide an insight into the role of each TLR molecule on the modulation of JE, as well as its mechanism of neuroinflammation control during JE progression.

## Introduction

Due to rapid changes in climate and demography, vector-transmitted arboviral diseases pose an increasing threat to global health and welfare [1]–[3]. Among the most severe arboviral infections known to affect the human race are those caused by members of the Flavivirus genus of the *Flaviviridae*. As such, flaviviruses, including Japanese encephalitis (JE), West Nile (WN), dengue, and tick-borne encephalitis virus (TBEV), are major emerging human pathogens, affecting millions of individuals worldwide. In addition, neurological disease frequently occurs upon infection with emerging flaviviruses, such as JEV, WNV, and TBEV [1]–[3]. Among neurotrophic flaviviruses, JEV is the most prevalent cause of viral encephalitis in the world, with approximately 67,900 cases reported annually [4]. Of these cases, about 25–30% are fatal and 50% result in permanent neuropsychiatric sequelae [4], for which JE is considered to be more fatal than West Nile encephalitis resulting in a fatality of 3–5% (1,100 death/29,000 symptomatic infection) [5]. Indeed, more than 60% of the world's population inhabit JE endemic areas which include eastern and southern Asia, and the virus is currently spreading to previously unaffected regions, such as Indonesia, Pakistan, and the northern area of Australia [6], [7].

Considerable progress in understanding the kinetics and mechanisms of JEV dissemination and pathogenesis has been made in murine models [1]–[3]. However, the molecular pathogenesis of JE still remains elusive. After peripheral amplification of the virus in dendritic cells (DC) and macrophages as primary target cells, the virus gains entry into the CNS through blood-brain barrier (BBB). While JEV infects and kills neurons directly [8], viral replication within microglia/glia and infiltrated monocytes leads to indirect neuronal killing *via* the secretion of pro-inflammatory cytokines (such as IL-6 and TNF-α) and soluble mediators which cause neuronal death [9]. Thus, it is believed that uncontrolled over-activation of microglia/glia and infiltrated monocytes during JE progression is one of the key factors in indirect neuronal cell death [9]. JEV-specific T cells and virus-neutralizing IgM and IgG are considered in part to play a role in the clearance of virus from peripheral lymphoid tissues, as well as from the CNS [7]. However, innate immune responses are considered to play a more crucial role in the early control of JEV infection due to delayed establishment of adaptive immunity, and may also be responsible for generating pathological levels of inflammation. Type I IFN gene expression and signaling are essential components of innate immune programs and control various viral infections, and thus may be potentially required for host control of JEV infection [10]–[13]. Studies in genetically deficient models suggest that type I IFN production after WNV infection is triggered by recognition of viral pathogenic-associated molecular patterns (PAMPs) through cytoplasmic helicases RIG-I and MDA5 as host PRRs [14]–[16], and thus the ablation of these molecules, their downstream signaling molecules (IPS-1), or transcriptional activators (IRF-3 and IRF-7) results in a greatly enhanced susceptibility to WNV infection [14]–[16]. However, type I IFN innate responses have also evolved through the recognition of membrane-bound cell-surface or intracellular Toll-like receptors (TLRs) [17], [18]. While RIG-I and MDA5 helicases recognize single- and double-stranded RNA in the cytosol and signal through IPS-1, TLRs on the cell surface or within endosomes recognize single- and double-stranded RNA and viral components, and subsequently transmit intracellular signals through adaptor molecules MyD88 and/or TRIF. The role of TLR signal pathway through MyD88 and/or TRIF in restricting flaviviral infection, as well as in modulating immune responses, remains less clear because of conflicting and intricate data [19]–[21]. Moreover, the impact of each TLR signal pathway on JE progression has not been addressed to date. We therefore became interested in identifying the key TLR molecule(s) which regulate JEV-induced neurological disease.

TLRs function as intermediates by interacting with products of viral replication, and transmitting signals to a cascade of adaptors and kinases that ultimately lead to the activation of transcription of cytokines and type I IFN genes. TLR3 recruits the adaptor molecule TRIF to induce type I IFN gene *via* interactions with TRAF3, TBK1, and IKKε, which, in turn, activate the latent transcription factors IRF-3 and IRF-7, whereas other TLRs associating with the adaptor protein MyD88 form a complex with TRAF6, IRAK1, and IRAK4 to activate kinases that regulate IRF-5 and IRF-7. Notably, TLR4 signal pathway uses both adaptor molecules MyD88 and TRIF to initiate the production of cytokine and type I IFN proteins. In viral infection, four TLRs, including TLR3, TLR7, TLR8 and TLR9, seem to play critical roles in the recognition of viral nucleic acid components, and TLR2 and TLR4 were shown to detect viral components such as envelope glycoproteins [22]–[26]. We have previously shown that JEV can modulate innate immune responses and subsequent adaptive responses in MyD88-dependent and independent pathways [27], which indicate that JEV may be recognized by certain TLR signal pathways, thereby affecting the outcome of JEV-induced neurological diseases. Therefore, we aimed to determine whether each TLR signal pathway modulated neurological disease caused by JEV infection, using several TLR-deficient mice (TLR2, TLR3, TLR4, TLR7, TLR9). Surprisingly, among the tested TLR-deficient mouse strains we found a contrasting result in TLR3^−/−^ and TLR4^−/−^ mice, *i.e.* TLR3^−/−^ mice were highly susceptible to JE, whereas TLR4^−/−^ mice showed markedly enhanced resistance to JE. Subsequently, we investigated the pathologic feature, type I IFN innate and adaptive immunity of TLR3^−/−^ and TLR4^−/−^ mice during JE progression. TLR3^−/−^ mice displayed severe neuroinflammatory reactions as well as enhanced BBB permeability by failure of the early control of viral replication, whereas TLR4^−/−^ mice elicited the effective regulation of viral replication and subsequent inflammatory reaction by inducing potent type I IFN innate immune responses against JEV. Notably, our data revealed that TLR4 ablation provided potent type I IFN innate responses through enhanced induction of antiviral ISG genes by alternative activation of IRF-3 and NF-κB in myeloid-derived DCs and macrophages. Also, TLR4^−/−^ mice showed an alteration of plasmacytoid DC subpopulation and CD4^+^Foxp3^+^ regulatory T cells, which were closely associated with enhanced type I IFN innate immune and JEV-specific CD4^+^ and CD8^+^ T cell responses. These results suggest that the balanced triggering of TLR array during JE progression plays a pivotal role in predicting the outcome of neurological disease.

## Results

### Contrasting regulation of JE by triggering TLR3 and TLR4 signal pathway

**Figure 1:**
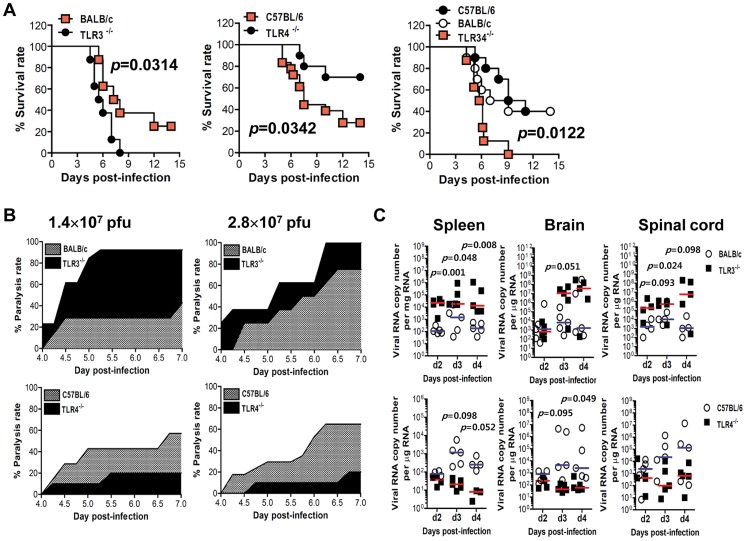
Contrasting regulation of JE by triggering TLR3 and TLR4 signal pathway. **A.** Susceptibility of TLR3^−/−^, TLR4^−/−^, and TLR3/4^−/−^ mice to JE. Four- to five-week-old mice (*n* = 10–18) were inoculated with JEV (2.8×10^7^ pfu), and the survival rate was examined over 15 days. **B.** Ratio of mice showing neurologic disorder during JE progression. Mice infected with JEV were examined every 6 h from 4 to 7 days pi. **C.** Viral burden in lymphoid and inflammatory tissues during JE progression. Viral burden in spleen, brain, and spinal cord of mice infected with JEV was assessed by real-time qRT-PCR at the indicated days pi. The viral RNA load was expressed by viral RNA copy number per microgram of total RNA (*n* = 5). Each symbol represents the level of an individual mouse; horizontal line indicates the median of each group.

It is believed that intracellular signaling through TLRs regulates host responses against various bacterial and viral infections. However, to date, the impact of TLR signaling on JEV-induced neuroinflammatory diseases and how this response is propagated and regulates *in vivo* innate and adaptive immunity have not been defined. To this end, we assessed the impact of each TLR molecule on JE, by evaluating the susceptibility of TLR2^−/−^, TLR3^−/−^, TLR4^−/−^, TLR7^−/−^, and TLR9^−/−^ mice to JEV infection (1.4×10^7^ pfu) (**Figure S1**). The ablation of TLR2, TLR7, and TLR9 molecules did not significantly affect the progression of encephalitis caused by JEV. However, somewhat surprisingly, TLR3- and TLR4- triggered molecular signaling pathways were observed to induce a completely contrasting regulation of JE. While all TLR3^−/−^ mice succumbed to neuroinflammatory diseases caused by JEV infection (*p* = 0.0153), TLR4^−/−^ mice showed enhanced resistance to JE, compared to wild-type mice (*p* = 0.0819). This contrasting regulation of JE by TLR3 and TLR4 molecules was more apparent (*p* = 0.0314 for TLR3 and *p* = 0.0342 for TLR4), when we evaluated the susceptibility of TLR3^−/−^ and TLR4^−/−^ mice to neuroinflammatory diseases after infection with a higher dose of JEV (2.8×10^7^ pfu) (**Figure 1A**). Also, the ablation of both TLR3 and TLR4 molecules induced a highly increased susceptibility to encephalitis caused by JEV infection (*p* = 0.0122). Likewise, TLR3^−/−^ mice infected with JEV showed more rapid signs of neurological disorder starting from 3 days pi, whereas TLR4^−/−^ mice showed delayed signs of neurological disorder with a lower frequency of occurrence, compared to wild-type mice (**Figure 1B**). To further examine the contrasting roles of TLR3 and TLR4 molecules, we assessed viral burden within lymphoid and the CNS tissues (**Figure 1C**). TLR3^−/−^ mice were found to exhibit 100–1,000-fold elevated viral load in spleen, brain, and spinal cord, but TLR4^−/−^ mice retained significantly lower viral loads with 10–100-fold decreased levels in the spleen, brain, and spinal cord, compared to those of wild-type mice. In addition, since two genetic backgrounds of mouse strains used for TLR3^−/−^ and TLR4^−/−^ mice could complicate the comparison of susceptibility to JE, we directly compared the susceptibility to JE between TLR3^−/−^ mice and wild-type mice, using TLR3^−/−^ mice derived from the same genetic background (H-2^b^) as TLR4^−/−^ mice. As expected, all TLR3^−/−^ (H-2^b^) mice succumbed to JE after infection with two different doses of JEV (1.4×10^7^ and 2.8×10^7^ pfu), while wild-type (H-2^b^) mice showed similar 50% and 70% mortality to wild-type mice of mouse strain (H-2^d^) used for TLR3^−/−^ mice, respectively (**Figure S2A** and **B**). This indicates that the genetic background of mouse strains used in this study did not affect the progression of neuroinflammation caused by JEV infection. Also, TLR3^−/−^ (H-2^b^) mice showed faster neurological disorder and severely reduced body weight by JEV infection. Supportively, TLR3^−/−^ (H-2^b^) mice retained higher viral burden within lymphoid and the CNS tissues (**Figure S2C**). Collectively, these results clearly indicate that triggering signal pathways through TLR3 and TL4 molecules differentially affect the outcome of neuroinflammatory disease caused by JEV infection and *in vivo* viral replication.

### TLR3, but not TLR4, is essential for the control of CNS inflammation following JEV infection

**Figure 2:**
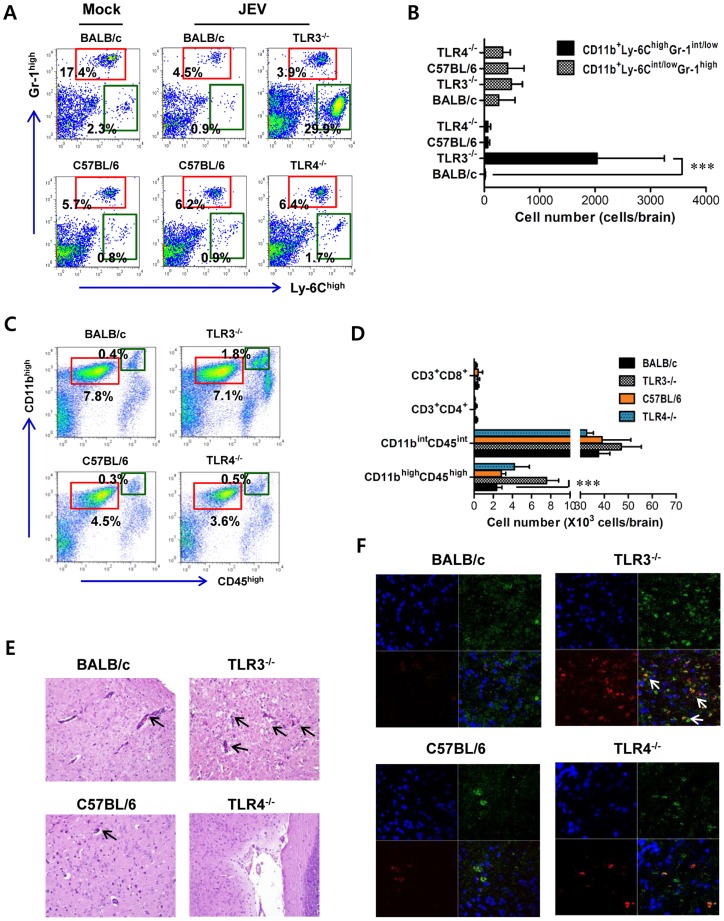
Enhanced inflammation of the CNS in TLR3^−/−^ mice following JEV infection. **A** and **B**. Early infiltration of inflammatory CD11b^+^Ly-6C^high^ monocytes. After heart perfusion at the 3rd day pi, the frequency (A) and absolute number (B) of CD11b^+^Ly-6C^high^ monocytes and CD11b^+^Gr-1^high^ granulocytes infiltrated into brain were analyzed by flow cytometric analysis. **C** and **D**. Percentage and number of resting microglia and activated microglia/macrophages. The frequency (C) and total number (D) of CD11b^int^CD45^int^ (resting microglia) and CD11b^high^CD45^high^ (activated microglia/macrophages) were determined at the 3rd day pi. The values in the representative dot-plot denote the average of the indicated cell population obtained from three individual experiment (*n* = 3–5). The bar in graph represents the average ± SD of the indicated cell number. **E**. H&E-stained brain tissue sections. Histological examinations were performed at the 4th day pi. The arrows denote the area of interest. **F**. Representative confocal microscopic images. Brain sections from TLR3^−/−^ and TLR4^−/−^ mice which were infected with JEV were co-stained for JEV antigen (red), the nuclear stain DAPI (blue), and the microglia/macrophage cell-specific marker CD11b (green) at 4 days pi. The data are representative of sections from at least five mice per group. ***, *p*<0.001 compared with the levels of the indicated group.

To further characterize the CNS inflammation caused by JEV infection, we assessed the infiltration of CD11b^+^Ly-6C^high^ cells into the CNS, as it has been demonstrated that CD11b^+^Ly-6C^high^ cells have properties of inflammatory monocytes [28]. Our results revealed that nearly identical percentage of CD11b^+^Gr-1^high^ neutrophil was retained in the brain of TLR3^−/−^ and wild-type mice 3 days following JEV infection, whereas a markedly higher frequency of infiltrated CD11b^+^Ly-6C^high^ monocytes in TLR3^−/−^ mice was observed with 10–20-fold increased levels 3 days after JEV infection, as compared to wild-type mice (**Figure 2A**). However, there were no significant changes in the proportion of CD11b^+^Gr-1^high^ neurophils and CD11b^+^Ly-6C^high^ inflammatory monocytes infiltrated in the brain of TLR4^−/−^ mice, following JEV infection. Also, the absolute number of inflammatory CD11b^+^Ly-6C^high^ monocytes infiltrated in the brain of TLR3^−/−^ mice increased 100–200-fold, whereas TLR4^−/−^ mice showed no significant changes in the absolute number of infiltrated monocytes or neutrophils following JEV infection (**Figure 2B**). To further determine whether the activation of infiltrated CD11b^+^Ly-6C^high^ monocytes could be affected by the ablation of TLR3 and TLR4 molecules, we characterized the phenotypes of infiltrated CD11b^+^Ly-6C^high^ monocytes. However, we found that there were no significant changes in phenotypic levels (CD40, CD80, CD86, MHC I, MHC II, F4/80) of brain infiltrated CD11b^+^Ly-6C^high^ monocytes between TLR3^−/−^ and TLR4^−/−^ mice (data not shown). It has been shown that microglia cells contribute to the pathogenesis of encephalitis caused by some neurotrophic viruses such as WNV [28], [29]. Thus, triple-color staining (CD11c/CD11b/CD45) was used to distinguish the resting and activated microglia. Based on the CNS myeloid cell classification of Ford et al. [30], equivalent percentages and absolute numbers of resting microglia (CD11b^int^CD45^int^CD11c^−^) were observed in brains of TLR3^−/−^ and TLR4^−/−^ mice following JEV infection. However, the frequency and absolute number of activated microglia (CD11b^high^CD45^high^CD11c^−^) were increased 4–5-fold in TLR3^−/−^ mice (**Figure 2C and D**). To confirm the effect of TLR3 and TLR4 molecules on patterns of leukocyte accumulation within the CNS, histological and confocal examinations were performed. Histological examination revealed that increased BBB permeability in JEV-infected TLR3^−/−^ mice was associated with perivascular cuffing, while JEV infection of TLR4^−/−^ mice elicited reduced numbers of infiltrating foci (**Figure 2E**). Similarly, significantly higher numbers of infiltrated CD11b^+^ cells were detected in TLR3^−/−^ mice by confocal microscopy, and a small subset of CD11b^+^ myeloid cells co-stained positive with JEV antigen (**Figure 2F**). Taken together, these results demonstrate that TLR3-induced signal pathway is essential for the control of neuroinflammation caused by JEV infection, while TLR4 molecules may be dispensable to provide resistance to fatal encephalitis.

**Figure 3:**
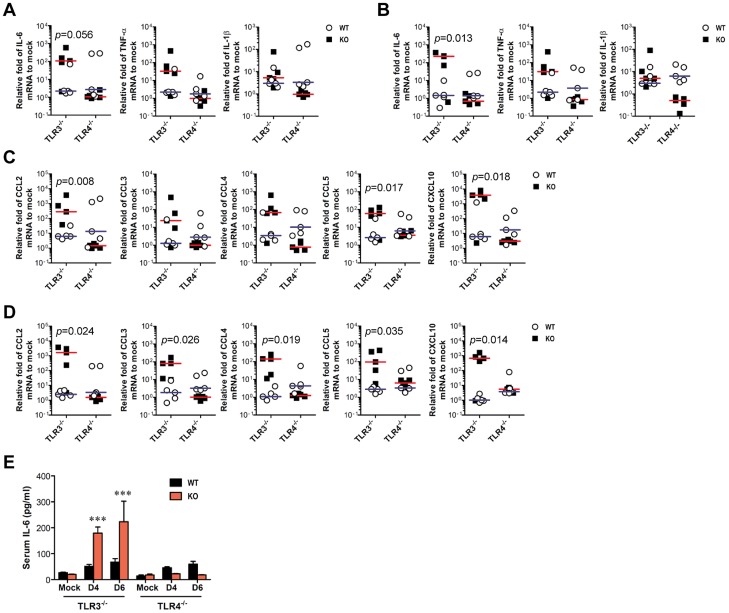
TLR3 ablation induces huge production of pro-inflammatory cytokines in inflammatory tissues. **A–D**. The expression of pro-inflammatory cytokine and chemokine in inflammatory tissues. The expression of pro-inflammatory cytokines and chemokines in brain (A and C) and spinal cord (B and D) was determined by real-time qRT-PCR 4 days pi. Each symbol represents the level of an individual mouse; horizontal line indicates median of each group. **E**. Systemic production of pro-inflammatory IL-6 cytokine. The levels of IL-6 in sera were determined by cytokine ELISA at the indicated day pi. Data represent the average ± SD derived from at least five mice per group. ***, *p*<0.001 compared with the levels of the wild-type mice.

In terms of severe neuroinflammation in TLR3^−/−^ mice, the expression levels of cytokines and chemokines within the CNS can be required for further explain encephalitis, because encephalitis caused by neurotrophic viruses is indirectly derived from CNS degeneration caused by robust immunological responses, such as the uncontrolled secretion of cytokines and chemokines, and resultant activation of microglia and astrocytes [7]–[9]. Therefore, we examined the expression of cytokines and chemokines in inflammatory tissues. We found that JEV infection of TLR3^−/−^ mice induced a highly enhanced expression of IL-6 and TNF-α in the CNS, including brain and spinal cord, whereas moderate changes in the expression of pro-inflammatory cytokines were observed in TLR4^−/−^ mice (**Figure 3A and B**). Also, the expression levels of chemokines including CCL2, CCL3, CCL4, CCL5, and CXCL10, which are involved in the migration of leukocytes into the CNS, was increased 10–1,000-fold in the brain and spinal cord of TLR3^−/−^ mice (**Figure 3C and D**). To further characterize how TLR3 and TLR4 molecules modulate the inflammatory reaction to JEV infection, we measured the levels of systemic IL-6 in serum of JEV-infected mice at 4 and 6 days pi. A trend towards more rapid induction and increased levels of IL-6 were observed in serum of TLR3^−/−^ mice compared to those of the wild-type mice (**Figure 3E**). However, no detectable differences in serum TNF-α levels were observed in TLR3^−/−^ or wild-type mice, since all samples clustered near the limit of detection by ELISA. Also, it was note worthy that TLR4 ablation induced no significant induction of systemic IL-6 and TNF-α. These results demonstrate that in the absence of TLR3, but not TLR4 molecules, greater pro-inflammatory cytokine and chemokine responses are induced during JE progression.

### TLR3, but not TLR4, regulates JEV-induced BBB disintegrity

**Figure 4:**
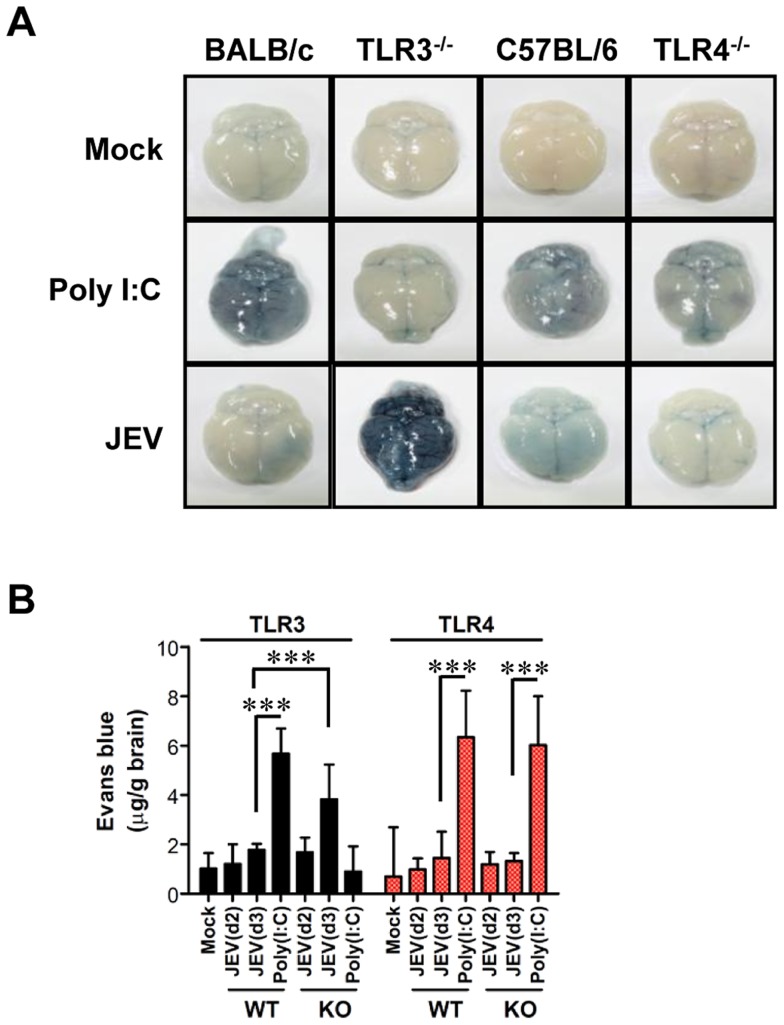
BBB permeability is increased after JEV infection in TLR3^−/−^ but not TLR4^−/−^ mice. TLR3^−/−^ and TLR4^−/−^ mice were given 1% Evans blue dye solution 2 and 3 days pi, and BBB permeability was evaluated by visualizing and quantifying extravasated Evans blue dye following vigorous heart perfusion. Mice injected with poly(I:C) were used as a positive control. **A**. Picture of extravasated Evans blue staining of whole brain 3 days pi. **B**. The amount of Evans blue dye diffused into whole brain. The amount of Evans blue dye was quantified by measuring the absorbance after tissue homogenization and precipitation. Data represent the average ± SD derived from six to eight mice per group. ***, *p*<0.001 compared with the levels of the indicated group.

Since BBB integrity is known to be damaged by neurotrophic virus-induced inflammation, such as WNV infection [20], [21], we assessed whether the ablation of TLR3 and TLR4 molecules could modulate BBB permeability and, possibly, allow for the earlier entry of virus and leukocytes within the CNS. Changes in BBB integrity over time following JEV infection, as revealed by extravasated Evans blue dye, showed that JEV infection of TLR3^−/−^ mice gave rise to increased BBB permeability 3 days pi (**Figure 4A**). In contrast, TLR4^−/−^ mice showed no significant change in BBB permeability following JEV infection. Supportively, the ablation of TLR3 molecules was found to induce increased BBB permeability by JEV infection, when the amount of extravasated Evans blue dye within the brain was measured by photometric analysis (**Figure 4B**). Notably, TLR3^−/−^ mice apparently retained increased amounts of extravasated Evans blue dye in the brain 3 days pi, compared to those of wild-type mice. This demonstrates that the ablation of TLR3, but not TLR4 molecule, is able to regulate BBB integrity following JEV infection.

### The spread of JEV in the brain of TLR3^−/−^ and TLR4^−/−^ mice after intracranial inoculation

**Figure 5:**
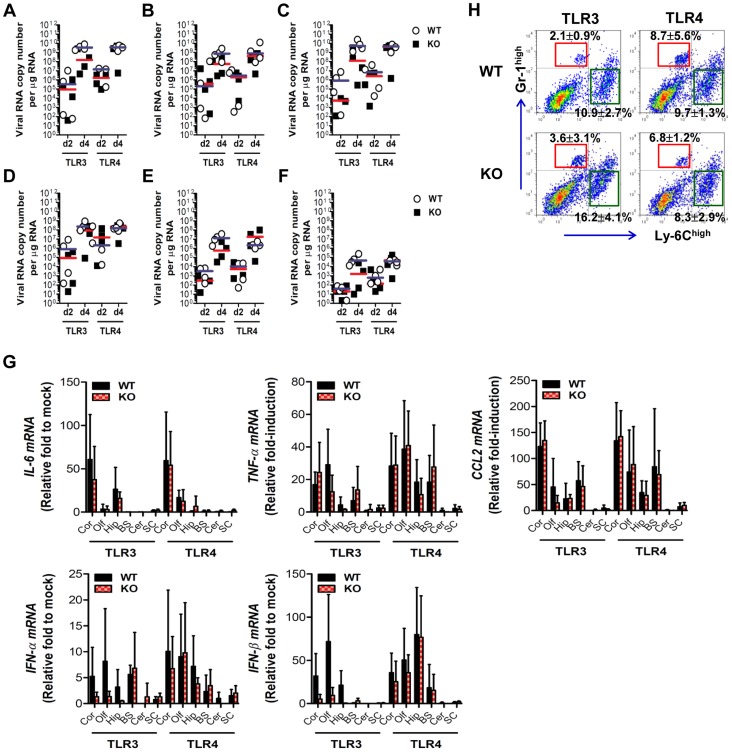
The spread of JEV in the brain of TLR3^−/−^ and TLR4^−/−^ mice after intracranial inoculation. TLR3^−/−^ and TLR4^−/−^ mice were inoculated with JEV (10^3^ pfu) by intracranial injection. Brains were harvested on days 2 and 4 pi, and then used for the determination of viral spread and cytokine expression. **A–F**. Viral burden in each sub-tissue of brain. The CNS tissues were separated into cortex (A), olfactory bulb (B), hippocampus (C), brain stem (D), cerebellum (E), and spinal cord (F). Viral burden was determined by real-time qRT-PCR. The viral RNA load was expressed by viral RNA copy number per microgram of total RNA. Each symbol represents the level of an individual mouse; the horizontal line indicates the median of each group. **G**. The expression levels of pro-inflammatory cytokine and type I IFN genes in each sub-tissue of brain. The expression levels were expressed by the indicated target gene levels relative to those in the mock-infected group. The bar represents the average ± SD of the indicated target gene levels obtained from each group (*n* = 5). Cor, cortex; Olf, olfactory bulb; Hip, hippocampus; BS, brain stem; Cer, cerebellum; SC, spinal cord. **H**. Leukocyte accumulation in the CNS of TLR3^−/−^ and TLR4^−/−^ mice after intracranial infection of JEV. TLR3^−/−^ and TLR4^−/−^ mice were inoculated with JEV (10^3^ pfu) by intracranial injection, and brains were harvested on day 2. Leukocyte populations were isolated by vigorous heart perfusion and then determined by flow cytometric analysis. The values in the representative dot-plots denote the average of the indicated cell population obtained from three individual experiments.

TLR3^−/−^ and TLR4^−/−^ mice showed distinct viral burdens in the CNS, which were closely associated with lethality to JE. This phenotype could be due to differential dissemination from the periphery and/or an independent antiviral effect in the CNS. To test this, wild-type, TLR3^−/−^, and TLR4^−/−^ mice were inoculated with 10^3^ pfu of JEV directly into the cerebral cortex *via* the intracranial (IC) route, and viral burdens in sub-tissues of brain (cortex, olfactory bulb, hippocampus, brain stem, cerebellum, and spinal cord) were monitored (**Figure 5A–F**). Wild-type as well as TLR3^−/−^ and TLR4^−/−^ mice showed rapid and complete mortality following IC infection of JEV, and there was no significant difference in the average survival time between wild-type and KO mice following IC infection of JEV (data not shown). Interestingly, TLR3^−/−^ and TLR4^−/−^ mice showed slightly lower levels of median viral burden in several sub-tissues of the brain. These data suggest that TLR3 and TLR4 molecules had no regulatory function on viral dissemination within the CNS following introduction, but appeared to have a subtle role in regulating viral replication in the CNS. In addition, we examined the expression of pro-inflammatory cytokine (IL-6 and TNF-α), chemokine (CCL2), and type I IFN (IFN-α and IFN-β). The expression of such cytokines in sub-tissues of brain following IC infection of JEV was consistently the same between wild-type and KO mice (**Figure 5G**). The accumulation of CD11b^+^Ly-6C^high^ leukocytes in the brain was slightly, but not significantly, higher in TLR3^−/−^ mice following IC infection of JEV, as compared to wild-type mice, and TLR4^−/−^ mice showed no significant change in leukocyte accumulation by IC infection of JEV (**Figure 5H**). Collectively, these results imply that TLR3 and TLR4 molecules have different roles in controlling the dissemination of JEV from the periphery into the CNS, rather than a regulatory role(s) on viral dissemination within the CNS after CNS invasion.

### Type I IFN responses are not blunted in TLR3^−/−^ mice following JEV infection

**Figure 6:**
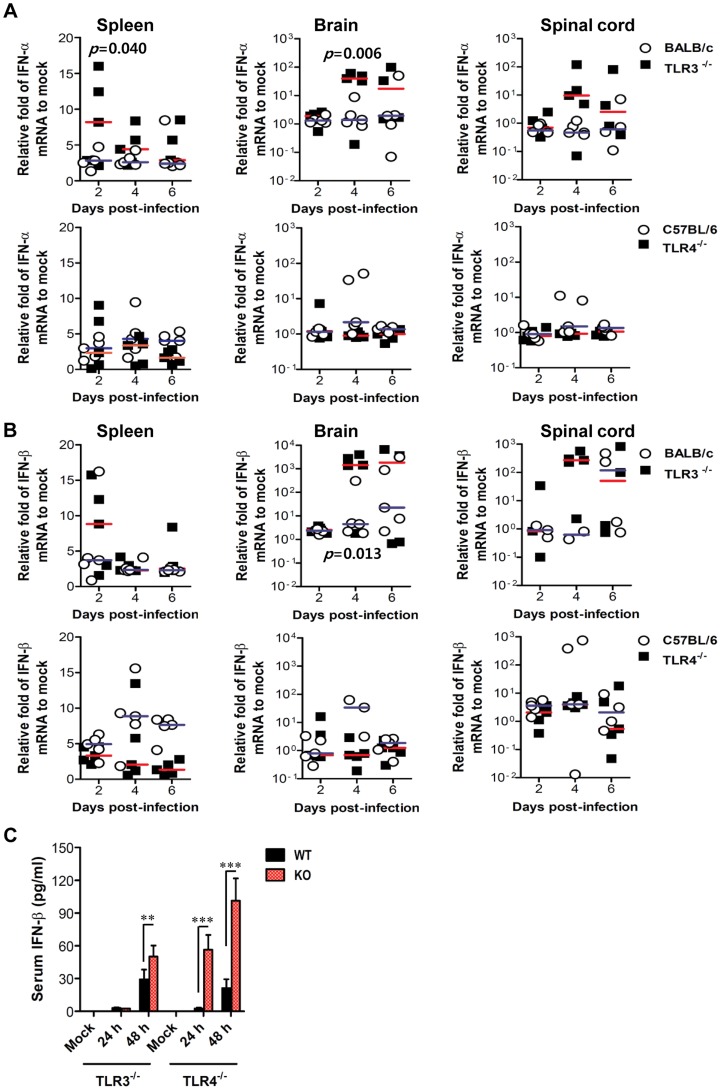
Localized and systemic type I IFN responses of TLR3^−/−^ and TLR4^−/−^ mice following JEV infection. **A** and **B**. The expression of type I IFNs (IFN-α and β) in lymphoid and inflammatory tissues. The levels of type I IFN (IFN-α and β) mRNA were determined by real-time qRT-PCR at the indicated day pi. Each symbol represents the level of an individual mouse; horizontal line indicates the median of each group. *p*-values were calculated using Student's t-test. **C**. Systemic IFN-β levels. The amount of serum IFN-β was determined by ELISA. Data represent the average ± SD derived from at least five mice per group. **, *p*<0.01; ***, *p*<0.001 compared with the levels of the indicated group.

It has been demonstrated that TLR3 molecules, in concert with RIG-I, MDA5, and TLR7, recognize viral RNA and induce type I IFNs through activation of adaptor molecule TRIF and subsequent transcription regulators IRF-3 and IRF-7. Also, triggering signal pathway by TLR4 molecule can activate IRF-3, IRF-5, and IRF-7 through adaptor molecules TRIF and MyD88, thereby inducing the production of type I IFNs (IFN-α and β) [31]–[33]. Therefore, since TLR3 and TLR4 molecules contribute to the generation of a normal IFN response through activation of IRF-3, IRF-5, and IRF-7 after infection with neurotrophic virus [14], [15], we tested whether the ablation of TLR3 and TLR4 molecules affected type I innate responses in JEV infection. Our data revealed that the expressions of IFN-α and β mRNA were increased in inflammatory and lymphoid tissues of TLR3^−/−^ mice with the levels peaked at 4 days pi, compared to those of wild-type mice (**Figure 6A and B**). In contrast, TLR4^−/−^ mice showed no significant increase of IFN-α or β mRNA expression in inflammatory or lymphoid tissues after JEV infection, compared to wild-type mice. Thus, the expression of type I IFN mRNA was not blunted in lymphoid and inflammatory tissues of TLR3^−/−^ mice following JEV infection, which indicates that alternate signal pathways *via* innate immune receptors, such as TLR7, RIG-I, and MDA5, can contribute to type I IFN responses in the absence of the TLR3 molecule. Similarly, TLR3^−/−^ mice showed delayed but slightly increased production of systemic IFN-β with peak levels attained at 48 h pi, compared to those of wild-type mice (**Figure 6C**). However, paradoxically and surprisingly, TLR4 ablation induced more rapid and markedly increased production of systemic IFN-β in serum, as compared to production rates in TLR3^−/−^ or wild-type mice. This result indicates that a deficiency of TLR4 molecules can modify the systemic production of type I IFNs. Importantly, it is worthy to note that this markedly enhanced production of systemic type I IFN-β protein in TLR4^−/−^ mice might contribute to the early control of viral replication in the periphery, thereby ultimately preventing viral dissemination into the CNS.

### Enhanced virus control and type I IFN responses in myeloid cells derived from TLR4^−/−^ mice after JEV infection

**Figure 7:**
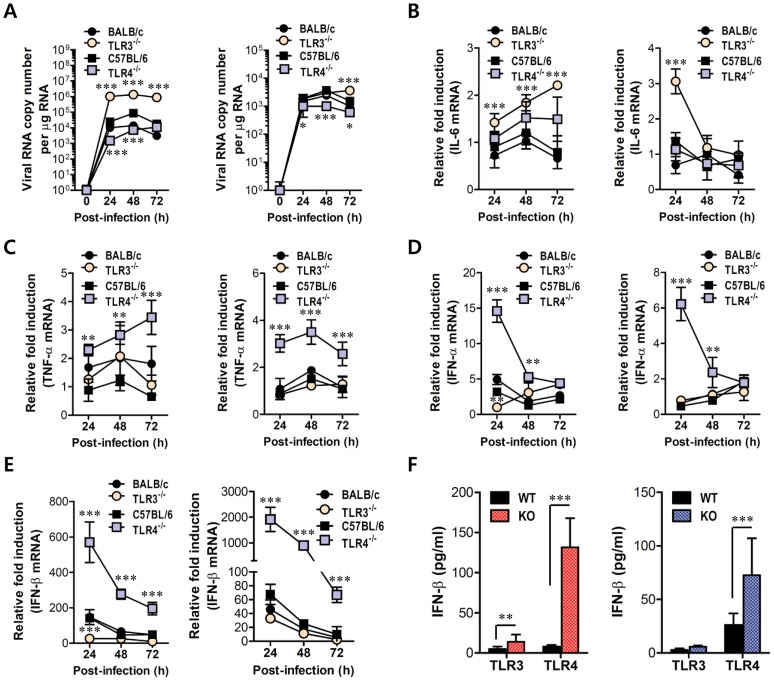
Virus control and type I IFN responses of myeloid cells derived from TLR3^−/−^ and TLR4^−/−^ mice to JEV infection. Primary bone marrow-derived DCs (BMDC) and macrophages (BMDM) recovered from TLR3^−/−^ and TLR4^−/−^ mice were infected with JEV at a MOI of 1.0 for viral replication and 10 for cytokine expression. **A**. JEV replication in BMDC and BMDM. Viral RNA replication was expressed by viral RNA copy number per microgram of total RNA. **B** and **C**. The expression of pro-inflammatory cytokines (IL-6 and TNF-α) in infected BMDC and BMDM. **D** and **E**. The expression of type I IFNs (IFN-α and β) in infected BMDC and BMDM. **F**. The secretion of IFN-β protein by infected BMDC and BMDM. The mRNA levels of the indicated cytokines were determined by real-time qRT-PCR and the cytokine levels in culture media were determined by ELISA. Data represent the average ± SD derived from BMDC and BMDM evaluated in quadruplicate. *, *p*<0.05; **, *p*<0.01; ***, *p*<0.001 compared with the levels of the wild-type control.

Myeloid cells, including tissue and lymphoid DCs and macrophages, are primary target cells of JEV infection and function to regulate the spread of virus to distant tissues such as the CNS [7]. Also, diverse cell populations can differentially utilize PRRs to induce innate immune responses upon viral infection. Therefore, these subtle functions may affect viral dissemination in the body and subsequent viral diseases. In addition, since our data showed that TLR4 ablation provided rapid and increased production of systemic IFN-β, we assessed whether TLR3 and TLR4 molecules affect JEV replication and type I IFN responses in myeloid-derived cells as primary target cells, in order to further define the differential roles of TLR3 and TLR4 molecules in controlling the progression of JE. Bone marrow-derived DCs (BMDC) and macrophages (BMDM) of TLR3^−/−^ and TLR4^−/−^ mice were infected with JEV and used to evaluate viral replication and the induction of pro-inflammatory cytokines and type I IFNs. TLR3^−/−^ BMDC sustained significantly higher JEV replication throughout the examination period compared to those of wild-type BMDC infected with JEV, whereas TLR4^−/−^ BMDC and BMDM showed delayed JEV replication at 24 and 48 h pi (**Figure 7A**). Also, a rapid and increased induction of IL-6 mRNA in TLR3^−/−^ BMDC and BMDM was observed, while TNF-α expression was increased 2-fold in JEV-infected TLR4^−/−^ BMDC and BMDM (**Figure 7B and C**), implying that the ablation of each TLR molecule could cause to trigger differential signal pathways to compensate for the production of pro-inflammatory cytokines. Surprising data was obtained from type I IFN innate responses of TLR4^−/−^ BMDC and BMDM after JEV infection. TLR4^−/−^ BMDC and BMDM induced rapid expressions of type I IFNs (IFN-α and β) mRNA with 10–100-fold increase in response to JEV infection, compared to wild-type BMDC and BMDM (**Figure 7D and E**). In contrast, IFN-α and β expression by TLR3^−/−^ BMDC and BMDM was virtually identical to those of wild-type BMDC and BMDM following JEV infection, except at an early time point (24 h pi), where levels were notably lower in TLR3^−/−^ BMDC. In support, TLR4^−/−^ BMDC and BMDM showed rapid secretion of IFN-β protein with 5–10-fold increase in response to JEV infection, while TLR3^−/−^ BMDC and BMDM showed slightly higher or identical levels of secreted IFN-β protein, compared to wild-type BMDC and BMDM (**Figure 7F**). Importantly, levels of IFN-β secretion in TLR3^−/−^ BMDC and BMDM were much lower than those of TLR4^−/−^ BMDC and BMDM. Conceivably, it is possible that potent type I IFN innate responses in TLR4^−/−^ myeloid-derived cells provides rapid and increased production of *in vivo* systemic type I IFNs, thereby contributing to the early control of viral replication in the absence of the TLR4 molecule. Collectively, these results indicate that TLR4 molecules are dispensable to induce rapid and increased response of type I IFN innate immunity in myeloid-derived cells upon JEV infection, and that a deficiency of TLR3 molecules does not virtually compromise type I IFN production in BMDC and BMDM after JEV infection.

### Enhanced induction of type I IFNs and ISGs in the absence of TLR4 is associated with alternative IRF3 phosphorylation and IκBα degradation in myeloid-derived DCs and macrophages

**Figure 8:**
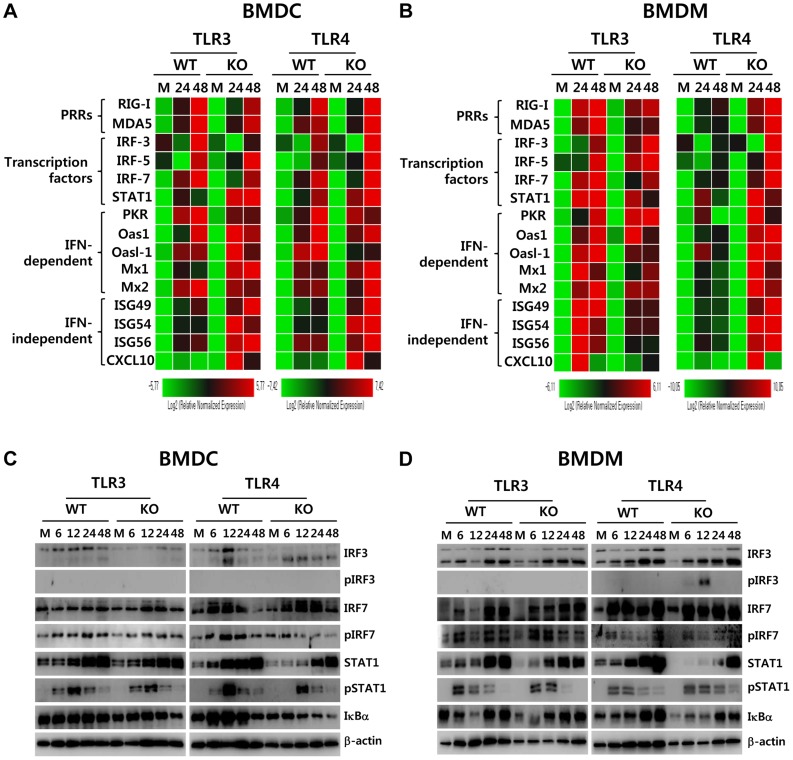
ISG induction, phosphorylation of IRFs, and IκBα degradation in primary myeloid cells derived from TLR3^−/−^ and TLR4^−/−^ mice after JEV infection. **A** and **B**. Clustered heatmap showing the expression of IRF, ISG, and RLR genes in infected BMDC and BMDM. Primary bone marrow-derived DCs (BMDC) and macrophages (BMDM) recovered from TLR3^−/−^ and TLR4^−/−^ mice were infected with JEV at a MOI of 10 or mock-infected (M), and employed to analyze the induction of IRF, ISG, and RLR genes at 24 and 48 h pi. The expression of each IRF, ISG, and RLR gene was normalized to β-actin after determining mRNA levels by real-time qRT-PCR, and displayed as the average of at least four independent samples, according to the indicated color on a log_2_ scale. **C** and **D**. Expression and phosphorylation of IRF3, IRF7, and STAT1 and IκBα degradation. BMDC and BMDM derived from TLR3^−/−^ and TLR4^−/−^ mice were infected with JEV at 10 MOI or mock-infected (M). At 6, 12, 24, and 48 h after infection, cells were lysed, separated by SDS-PAGE and analyzed by western blot to detect unphosphorylated and phosphorylated form of target proteins using specific Abs. One representative picture of at least three experiments is shown.

Since myeloid-derived DCs and macrophages of TLR4-ablated mice showed highly enhanced production and expression of antiviral type I IFNs upon JEV infection, we measured the induction levels of antiviral ISG genes to define this finding in greater detail. We specifically focused on PRRs (RIG-I [DDX1], MDA5 [IFITH1]), their transcription factors (IRF3, IRF5, IRF7), and IFNAR transcription factor (STAT1) as well as IFN-independent (ISG49 [IFIT3], ISG54 [IFIT2], ISG56 [IFIT1], CXCL10) and dependent genes (PKR, Mx1, Oas1, Oasl-1). Our results revealed that TLR3^−/−^ BMDC and BMDM showed differential responses of antiviral ISG expression upon JEV infection (**Figure 8A and B**). TLR3^−/−^ BMDC showed less induction of PRR genes (RIG-I and MDA5) and their transcription factors (IRF-3 and IRF-7), but member of genes (ISG49, ISG54, ISG56, CXCL10) that are induced in IFNAR^−/−^ cells (*i.e.*, are IFN-independent) [34], [35] were expressed in TLR3^−/−^ BMDC with slightly higher levels, compared to those of wild-type BMDC. This result was consistent with the fact that TLR3^−/−^ BMDC showed slightly higher or identical secretion of IFN-β compared to wild-type BMDC (**Figure 7F**), because IFN-independent ISG genes can also be induced through ISRE binding of ISGF3 complex initiated by type I IFN receptor [15]. In contrast, TLR3^−/−^ BMDM showed less induction of IFN-independent ISG genes (ISG49, ISG54, ISG56, CXCL10) as well as IFN-dependent ISG genes (PKR, Mx1, Mx2) and members of the 2′-5′-oligoadenylate synthetase family (Oas1, Oasl-1), compared to wild-type BMDM. This result implies that macrophages could be more compromised in the inductiveness of type I IFN innate responses than DCs, if the TLR3 molecule was ablated. The prominent induction of antiviral ISG genes was observed in TLR4^−/−^ BMDC and BMDM after JEV infection (**Figure 8A and B**). TLR4^−/−^ BMDC showed enhanced expression of PRR genes (MDA-5) and its transcription factors (IRF-3, IRF-5, IRF-7), and IFN-dependent genes (PKR, Oasl-1), as well as IFN-independent genes (ISG49, ISG 54, ISG 56, CXCL10). Also, TLR4^−/−^ BMDM showed much more apparently and highly induced expression of antiviral ISG genes after JEV infection compared to those of wild-type BMDM and other cells, because TLR4^−/−^ BMDM induced the expression of all tested ISG genes (PRRs, transcription factors, IFN-dependent and independent genes) with higher levels than other cells. Notably, TLR4^−/−^ BMDM showed markedly enhanced induction of both IFN-dependent (PKR, Oas1, Oasl-1, Mx1, Mx2) and independent genes (ISG49, ISG54, ISG56, CXCL10), compared to TLR3^−/−^ BMDM that showed less induction of such genes. Therefore, these results support that a deficiency of TLR4 molecule provides more efficient type I IFN innate immune responses in DCs and macrophages following JEV infection.

To further define the induction of antiviral IFN-independent and dependent ISG genes in JEV-infected TLR3^−/−^ and TLR4^−/−^ DCs and macrophages, the activation state of associated transcription factors was examined by western blot. In line with antiviral ISG induction data, TLR3^−/−^ BMDC displayed decreased expression of IRF-3 and IRF-7 at 6–48 h and 48 h pi, respectively, and phosphorylated form of IRF-3 was not detected in both TLR3^−/−^ and TLR4^−/−^ BMDC (**Figure 8C**), which supports that enhanced induction of antiviral IFN-independent ISG genes (ISG49, ISG54, ISG 56, CXCL10) in TLR3^−/−^ and TLR4^−/−^ BMDC may be caused by stimulation of IFNAR signal through increased IFN-β secretion [15]. Since slightly delayed phosphorylation of STAT1, an IFNAR transcription factor, was observed in TLR3^−/−^ and TLR4^−/−^ BMDC, other pathways to activate NF-κB were also considered to contribute to enhanced induction of IFN-independent ISG genes. Interestingly, this notion can be explained by the result that faster degradation of IκBα was detected in TLR4^−/−^ BMDC. IκBα proteins are phosphorylated *via* IκB kinase (IKK) activated by signal transducers, and are subsequently degraded after release of NF-κB [36]. Therefore, these results suggest that TLR4^−/−^ BMDC could have evolved as yet unknown pathway(s) to activate NF-κB upon JEV infection, thereby inducing enhanced expression of type I IFNs and ISG genes. In addition, somewhat interestingly, transiently phosphorylated form of IRF-3 was strongly detected in TLR4^−/−^ BMDM, but not TLR3^−/−^ BMDM, as early as 6 and 12 h pi (**Figure 8D**). Also, TLR4^−/−^ BMDM showed prolonged and strong phosphorylation of STAT1 after JEV infection, compared to wild-type BMDM. Therefore, it was considered that activation of IRF3 and STAT1 in TLR4^−/−^ BMDM derived potent type I IFN production as well as the induction of broad antiviral IFN-independent and IFN-dependent ISG genes.

### Induction of type I IFN and ISGs in primary cortical neurons derived from TLR3 and 4-deficient mice after JEV infection

**Figure 9:**
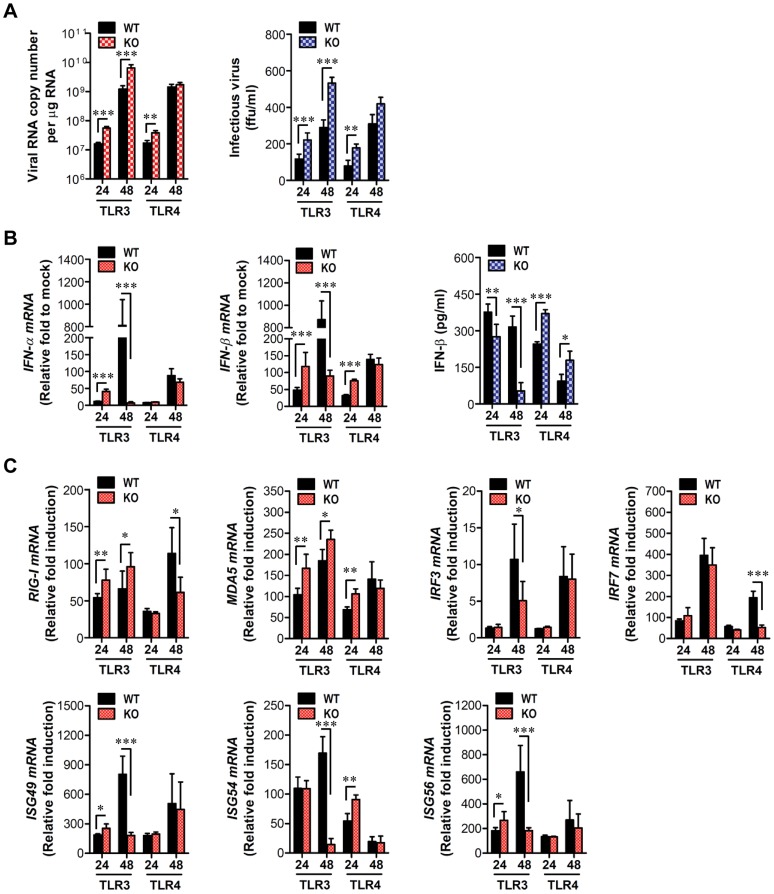
Induction of type I IFNs and ISGs in primary cortical neurons derived from TLR3^−/−^ and TLR4^−/−^ mice after JEV infection. Primary cortical neurons generated from TLR3^−/−^ and TLR4^−/−^ mice were infected at an MOI of 0.1, and viral replication and type I IFNs responses at 24 and 48 h pi were analyzed. **A**. JEV replication. JEV replication was determined by both real-time qRT-PCR and focus-forming assay. **B**. The expression of type I IFNs (IFN-α and IFN-β) mRNA and IFN-β secretion in primary cortical neurons infected by JEV. **C**. Induction of IRFs, ISGs, and RLRs gene in infected primary cortical neurons. The mRNA levels of the indicated gene were determined by real-time qRT-PCR, and IFN-β levels in culture media were determined by ELISA. Data represent the average ± SD derived from primary cortical neurons quadruplicate. *, *p*<0.05; **, *p*<0.01; ***, *p*<0.001 compared with the levels of the indicated group.

Neurons may be the main target cell of JEV infection in the CNS, and their death is a key factor in pathogenesis and neurological sequelae [8]. To examine whether TLR3 and TLR4 molecules can regulate JEV replication in neurons, primary cortical neurons generated from wild-type as well as TLR3^−/−^ and TLR4^−/−^ mice were infected with JEV, and virus yield, type I IFN responses and ISG expression were evaluated. It was likely that wild-type neurons were more permissive to JEV infection than DCs or macrophages, because infection of neurons with 10-fold less virus (MOI 0.1 versus 1.0) produced over ∼10^5^ viral RNA within 24 h (**Figure 7A** and **Figure 9A**). In the absence of TLR3 molecule, JEV replicated faster, resulting in a 1.5–2.0-fold increase in infectious virus production between 24 h and 48 h pi, as compared to infected wild-type neurons. The ablation of TLR4 molecule showed earlier replication of JEV at 24 h pi, but the levels of virus were similar in both wild-type and TLR4^−/−^ neurons at 48 h pi (**Figure 9A**). Biphasic type I IFN mRNA induction was observed, with slightly higher levels at 24 h pi but much lower at 48 h pi in TLR3^−/−^ neurons, compared to wild-type neurons (**Figure 9B**). In contrast, TLR4^−/−^ neurons showed transient induction of IFN-β at 24 h pi, after which IFN-β mRNA levels were comparable in both wild-type and TLR4^−/−^ neurons. The secretion of IFN-β protein in culture media was markedly lower in TLR3^−/−^ neurons at 48 h pi, while TLR4^−/−^ neurons showed increased expression and production of IFN-β at both 24 h and 48 h pi, as compared to those of wild-type neurons (**Figure 9B**). Also, it seemed that the expression of antiviral ISGs in TLR3^−/−^ neurons followed type I IFN responses; hence, ISG49 and ISG56 showed transient increases at 24 h pi but much lower expression at 48 h pi (**Figure 9C**). Also, a higher expression of RIG-I and MDA-5, a cytosolic PRRs of viral RNA, was observed in TLR3^−/−^ neurons, but their transcription factor IRF-3 was shown with decreased expression levels, as compared to wild-type neurons. It was thought that this caused the reduction in IFN-β production in TLR3^−/−^ neurons at 48 h pi. TLR4^−/−^ neurons showed transiently higher expression of ISG54 and MDA5 at 24 h pi, but the decreased levels of RIG-I and IRF-7 expression was observed at 48 h pi. Collectively, these results suggest that TLR3 may have an independent and subordinate role in triggering type I IFN innate responses in cortical neurons, because type I IFN responses and ISGs expression were much decreased at a later time point (48 h pi). Also, TLR4^−/−^ cortical neurons appeared to induce less potent type I IFN innate immune responses than TLR4^−/−^ DCs and macrophages, which indicates that specific types of cells differentially trigger innate immune responses following JEV infection.

### Enhanced type I IFN innate and JEV-specific CD4^+^/CD8^+^ T cell responses in TLR4^−/−^ mice are associated with altered number of plasmacytoid DCs and CD4^+^Foxp3^+^ Treg cells in lymphoid tissues

**Figure 10:**
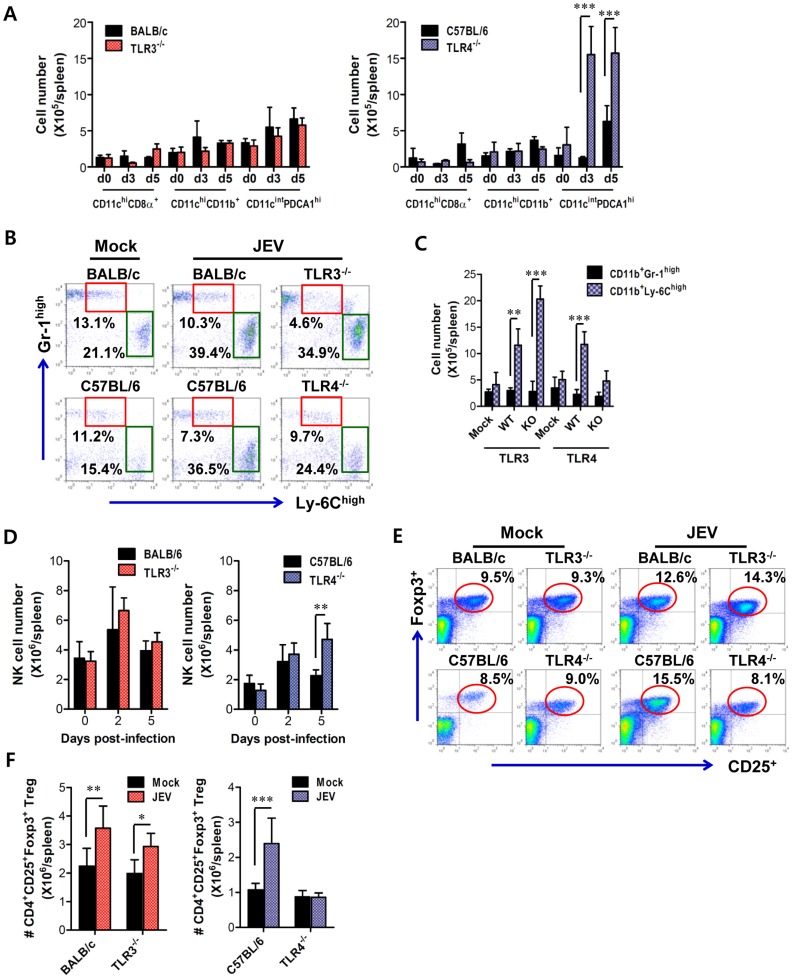
Alteration of myeloid-derived and immune cell subsets in lymphoid tissues of TLR3^−/−^ and TLR4^−/−^ mice during JE progression. **A**. Alteration of splenic DC subpopulation in TLR3^−/−^ and TLR4^−/−^ mice. The absolute numbers of conventional DCs (CD11c^high^CD11b^+^ and CD11c^high^CD8α^+^) and plasmacytoid DCs (CD11c^int^PDCA-1^high^) were determined at the indicated days pi. **B** and **C**. The frequency and absolute number of inflammatory CD11b^+^Ly-6C^high^ monocytes in the spleen of TLR3^−/−^ and TLR4^−/−^ mice. Inflammatory CD11b^+^Ly-6C^high^ monocytes were analyzed at the 3rd day pi. **D**. The absolute number of CD3^−^DX5^+^ NK cells in the spleen of TLR3^−/−^ and TLR4^−/−^ mice during JE progression. **E** and **F**. The frequency and absolute number of CD4^+^CD25^+^Foxp3^+^ Treg cells in the spleen of TLR3^−/−^ and TLR4^−/−^ mice. The proportion and total number of CD4^+^CD25^+^Foxp3^+^ Treg cells in the spleen of wild-type, TLR3^−/−^, and TLR4^−/−^ mice were determined by flow cytometric analysis at 7 days pi. The values in the representative dot-plots denote the average of the indicated cell populations obtained from three individual experiments (*n* = 3–4). The bar in graph represents the average ± SD of the total number of the indicated cell population. *, *p*<0.001; **, *p*<0.01; ***, *p*<0.05 compared with the levels of the indicated group.

TLR signal pathway through MyD88 and/or TRIF adaptor molecules is required in some cases for antigen-specific antibody responses [37], [38], which may contribute to the control of JEV dissemination and replication in the brain. Our data revealed that TLR3 ablation showed slightly, but not significantly, increased level of IgM and IgG, while TLR4^−/−^ mice showed significantly increased levels of JEV-specific IgM and IgG, compared to wild-type mice (**Figure S3A**). Also, JEV infection showed marginally increased numbers of CD4^+^, CD8^+^ T, and CD19^+^ B cells with activated phenotypes, as corroborated by the expression of surface markers, such as CD69, CD44, and CD80; however TLR3 and TLR4 molecules did not show apparently regulatory functions in T and B lymphocytes (**Table S1**). Since effector antigen-specific CD4^+^ and CD8^+^ T cell responses are also required for the control and clearance of JEV in the CNS as well as in peripheral tissues [7], we evaluated whether the ablation of TLR3 and TLR4 molecules altered JEV antigen-specific CD4^+^ and CD8^+^ T cell responses. A deficiency of TLR3 molecules resulted in a similar percentage and absolute number of CD4^+^ and CD8^+^ T cells expressing IFN-γ and TNF-α, whereas TLR4^−/−^ mice showed an increased percentage and absolute number of IFN-γ and TNF-α-producing CD4^+^ and CD8^+^ T cells (**Figure S3B–E**). Along with potent type I IFN innate responses, these data indicate that TLR4 ablation could provide enhanced antigen-specific responses, thereby contributing in part to the control of virus replication and dissemination. Therefore, to further characterize the immunological parameters associated with potent type I IFN innate and adaptive immune responses in JEV-infected TLR4^−/−^ mice, we analyzed the immune cellular components related to type I IFN innate and adaptive immune responses. TLR3^−/−^ and TLR4^−/−^ mice were challenged with JEV, and spleens were harvested at 3 and 5 days pi. At the early phase of infection, analysis of the spleen provides an insight into how TLR3 and TLR4 molecules modulate innate immune and inflammatory responses immediately after infection, because JEV was administered intraperitoneally. Analysis of lymphoid CD8α^+^ and myeloid CD11b^+^ DC subsets revealed that JEV-infected TLR3^−/−^ and TLR4^−/−^ mice exhibited similar increases in both DC subsets, compared to those of infected wild-type mice (**Figure 10A**). However, somewhat surprisingly, the ablation of TLR4 molecule resulted in a highly increased number of CD11c^int^PDCA-1^high^ plasmacytoid DC (pDC) subset, which is known as a potent cellular component to produce type I IFNs in response to viral infection [39]. Thus, it was considered that highly increased pDC number might contribute in part to enhanced production of systemic IFN-β in TLR4^−/−^ mice. TLR4^−/−^ mice also showed a decreased frequency of inflammatory CD11c^−^CD11b^+^Ly-6C^high^ monocytes and no significant changes in the absolute number, whereas a significant increased number, but not frequency, of inflammatory monocytes was observed in TLR3^−/−^ mice, compared to that in wild-type mice (**Figure 10B and C**). This implies that TLR4^−/−^ mice exhibit a mild inflammatory reaction in the spleen. In addition, a deficiency of TLR4 molecule provided an increased number of NK cells at 5 days pi, but TLR3 molecule had no modulatory function on NK cell number (**Figure 10D**). Moreover, since CD4^+^CD25^+^Foxp3^+^ Treg cells contribute to the dampening of innate and adaptive immune responses during acute viral infection [40], we addressed the frequency and number of CD4^+^CD25^+^Foxp3^+^ Treg cells in the spleen. We found that the frequency and absolute number of CD4^+^CD25^+^Foxp3^+^ Treg cells were increased 1.5–2-fold in response to JEV infection in wild-type mice (**Figure 10E and F**). TLR3^−/−^ mice showed identical increase of CD4^+^CD25^+^Foxp3^+^ Treg cells to wild-type mice, while in TLR4^−/−^ mice a reduced frequency and absolute number of CD4^+^CD25^+^Foxp3^+^ Treg cells was observed, which indicates that TLR4 molecule could be involved in the increase of CD4^+^CD25^+^Foxp3^+^ Treg cell numbers following JEV infection. Collectively, these results suggest that increased number of CD11c^int^PDCA-1^high^ pDC subpopulation and reduced CD4^+^CD25^+^Foxp3^+^ Treg cells are closely associated with enhanced type I IFN innate immunity and JEV-specific CD4^+^ and CD8^+^ T cell responses in TLR4^−/−^ mice.

## Discussion

Although recognition of ssRNA virus, such as flavivirus, *via* cytosolic helicase RIG-I and MDA5 may be dominant to induce type I IFN innate responses [14]–[16], the role of TLRs as first-front line of innate immune receptors in the extracellular space, including the cell membrane and endosome, remains still undefined in flaviviral infections, due to conflicting and intricate data. Furthermore, despite the pathological importance of JE as a major cause of acute encephalitis, the role of TLR signal pathways in JE progression has not been fully explored to date. Here, we observed strikingly contrasting regulation of JE *via* TLR3 and TLR4 signal pathways; TLR3 ablation elicited highly enhanced susceptibility to JE, whereas TLR4 ablation provided significantly enhanced resistance to JE rather than inducing increased susceptibility. In the present study, interesting clues to such contrasting regulation of JE by TLR3 and TLR4 molecules were derived from the differential induction of type I IFN innate responses in TLR3^−/−^ and TLR4^−/−^ mice. Notably, TLR4 ablation induced potent type I IFN innate responses through enhanced induction of antiviral ISG genes by alternative activation of IRF-3 and NF-κB in DCs and macrophages. Additionally, altered CD11c^int^PDCA-1^high^ pDC and CD4^+^CD25^+^Foxp3^+^ Treg number in TLR4^−/−^ mice appeared to contribute in part to enhanced type I IFN innate as well as JEV-specific T cell responses. Collectively, potent type I IFN innate and adaptive immune responses generated in peripheral lymphoid tissues after JEV infection were closely coupled with a reduced JE lethality in TLR4^−/−^ mice. These findings imply that the balanced triggering of TLR signal array by viral components during JE progression could be responsible for determining the outcome of disease through negative and positive regulatory factors.

There are several conflicting reports on the role of TLR3 signaling pathway in neurological diseases caused by viral infection [41], [42]. A deficiency of TLR3 in humans predisposes to a genetic risk factor for herpes simplex virus encephalitis [43] and influenza A virus-induced encephalopathy [44], but TLR3^−/−^ mice infected with influenza [45], punta toro [46], and vaccinia viruses [47] showed improved survival and decreased production of inflammatory cytokines. Strikingly conflicting role of TLR3 signal pathway was derived from an infection model with WNV [20], [21]. While TLR3 ablation protected mice from WNV lethal infection by decreased systemic TNF-α and IL-6 production and BBB permeability [20], there is a report demonstrating that TLR3 molecules are essential in protecting from WNV infection [21]. Our results favor the latter report. TLR3^−/−^ BMDC, but not to BMDM, showed defective type I IFN innate responses at an early time (24 h pi), which may allow early viral replication. This result is in contrast to that of WNV infection, where TLR3 molecule did not modulate WNV replication and IFN induction in primary myeloid cells [21]. Although TLR3^−/−^ BMDC is more permissive to JEV replication, JEV-infected TLR3^−/−^ BMDC elicited similar levels of type I IFN responses to wild-type BMDC with delayed kinetics, and TLR3^−/−^ mice also showed no blunted type I IFN responses in lymphoid and local tissues. This suggests that enhanced tissue tropism and rapid viral entry into the CNS is not affected by locally induced type I IFN responses. Type I IFN responses of TLR3^−/−^ mice were considered not to be attenuated since cytosolic RIG-I and MDA5 molecules are intact. However, TLR3 molecule appeared to play more important role in inducing type I IFN responses of neuron cells than BMDC and BMDM, because TLR3^−/−^ neuron cells showed a markedly reduced expression and production of type I IFN and ISGs at a late time (48 h pi), thereby promoting viral replication. This implies that TLR3 molecule had differential modulatory functions on type I IFN innate responses and JEV replication in a cell-type restricted manner. However, considering that TLR3^−/−^ and TLR4^−/−^ mice showed no difference in CNS replication of JEV following IC infection, subtle changes of CNS system, such as innate responses of microglia and astrocyte, appear to modulate the *in vivo* spread of directly inoculated JEV in the CNS.

Increased BBB permeability by systemic TNF-α and IL-6 appears to promote an earlier entry of virus into the CNS. In contrast to WNV infection, where TLR3^−/−^ mice showed no change in BBB permeability [21], TLR3^−/−^ mice, but not TLR4^−/−^ mice, elicited increased BBB permeability associated with a huge production of systemic IL-6. Also, this result was in contrast with a previous study in which TLR3^−/−^ mice showed reduced cytokine (e.g., TNF-α and IL-6) responses, BBB permeability, neuroinvasion, and mortality following infection with mammalian cell-passaged WNV [20]. Nonetheless, our results showed some similarities with previous reports using WNV, such as increased viral burden in peripheral tissues. Although the impact of TLR3 molecule on BBB permeability is likely to differ, depending on the context and details of the model, virus-culture conditions, and the viral strain being tested, the failure of early viral clearance in the periphery of TLR3^−/−^ mice may ultimately cause enhanced inflammatory reactions, thereby increasing BBB permeability and viral load in the CNS. One intriguing result in this study was that TLR7^−/−^ mice showed no change in susceptibility to JE, since TLR7 molecule can recognize ssRNA of JEV. This result was in contrast with the report that the TLR7 molecule is involved in modulating the progression of WNV encephalitis *via* an IL-23-dependent accumulation of leukocytes in the CNS [48]. Although systemic levels of proinflammatory cytokines and type I IFNs were higher in TLR7^−/−^ than in wild-type mice [48], it is expected that splenic pDC or circulating pDC from TLR3^−/−^ mice may also have contributed to the type I IFN responses, because TLR7 signal pathway was intact in TLR3^−/−^ mice. In addition, we previously found that TLR2 molecule had modulatory function in cross-presentation of OVA protein using JEV-infected TLR2^−/−^ mice, suggesting that JEV infection may be also be recognized by TLR2 molecule [49]. However, in this study, TLR2 signal pathway had no impact on the progression of JE. One trivial explanation of this result is that TLR2 signal pathway was not involved in inducing pathologic disease by JEV infection, no matter what OVA cross-presentation is regulated by JEV infection in a TLR2-dependent manner.

The most intriguing result in this study was that TLR4^−/−^ mice showed markedly enhanced resistance to JE. To date, the role of TLR4 signal pathway in inducing innate and adaptive immune response against JEV and other flaviviruses has not been defined. Our results demonstrate that TLR4 ablation strongly induces *in vivo* systemic type I IFN innate responses, as well as type I IFN expression and production from myeloid-derived cells upon JEV infection. This presumably promotes early clearance of virus. In spite of the existence of TLR4 prototype ligand, LPS, a growing number of reports suggest that TLR4 molecule is biologically relevant, and is responsive to viral proteins, including those of Ebola virus [23], hepatitis C virus [24], and respiratory syncytial virus [25], leading to the induction of proinflammatory cytokines. We are not sure whether the induction of potent type I IFN innate responses in the absence of TLR4 signal pathway was mediated directly by enhanced signal transduction of other PRRs, such as TLR3, RIG-I, and MDA5, and/or indirectly by soluble factors produced from host cells by viral infection, i.e. DAMPs. However, our results provide one explanation as to how TLR4^−/−^ myeloid-derived cells induce potent type I IFN innate responses, *i.e.* enhanced activation of NF-κB through unknown pathway(s) in DCs, and transient activation of IRF3 at 6–12 h pi and prolonged activation of STAT1 in macrophages. The expression of antiviral ISG genes in myeloid-derived cells after JEV infection was induced by both direct (by IRF-3) and indirect (by IFN-β production and IFNAR signaling) pathways. Considering that only small faction (10–20%) of myeloid-derived cells is infected by JEV [49], uninfected myeloid-derived cells are thought to substantially contribute to antiviral ISG induction through stimulation of IFNAR signal after binding with secreted IFN-β proteins. This notion was supported by two results, *i.e.* 1) induction of IFN-dependent genes (PKR, Mx1, Oas1) in TLR3^−/−^ BMDC, TLR4^−/−^ BMDC and BMDM with increased secretion of IFN-β after JEV infection, and 2) no detection of phosphorylated IRF-3 except in TLR4^−/−^ BMDM. Also, transient activation of IRF3 and prolonged activation of STAT1 explains strong induction of both IFN-independent ISG (ISG49, ISG54, ISG56, CXCL10) and dependent genes (PKR, Oas1, Mx1, Mx2) in TLR4^−/−^ macrophages. Although NF-κB activation in DCs and IRF-3 and STAT1 activation in macrophages after JEV infection support potent type I IFN innate responses in the absence of TLR4 molecule, how these signal molecules are activated remains still undefined. Therefore, future studies will be required to delineate the mechanistic and functional intermediates that link and regulate NF-κB, IRF-3 and STAT1 signal pathway in the absence of TLR4 molecule.

In addition, our results is strengthened by a recent report that TLR4^−/−^ or TLR4 antagonist-treated mice are highly refractory to influenza-induced lethality, due to blocking inflammation by host-derived, oxidized phospholipid that potently stimulates TLR4 [50], [51]. One similarity with our data is that mice treated with TLR4 antagonist, Eritoran, or TLR4^−/−^ mice had reduced lung pathology to infection with influenza virus, which is characterized by the reduction of viral burden and proinflammatory cytokine expression. However, it is not certain whether Eritoran-treated or TLR4^−/−^ mice displayed rapid and enhanced type I IFN innate responses after infection with influenza virus. Thus, it is worthwhile identifying whether blocking TLR4 signal pathway by antagonists such as Eritoran, affects JE progression through the induction of potent type I IFN innate responses. This study will provide valuable insights into developing therapeutic strategies to viral encephalitis caused by neurotrophic virus such as JEV and WNV. Analogously, in the absence of TLR4 molecule, the enhanced expansion of CD11b^+^Ly-6C^high^ “inflammatory monocytes” was not observed in comparison with TLR3^−/−^ mice, which was suggestive that in TLR4^−/−^ mice mild inflammatory responses were elicited in the spleen. This monocyte subset migrates to the site of infection, secretes pro-inflammatory cytokines, and thereby exacerbates immunopathologic diseases [28]. Thus, the aberrant recruitment and expansion of these CD11b^+^Ly-6C^high^ inflammatory monocytes may also contribute to JE immunopathogenesis in TLR3^−/−^ mice.

The production and response of type I IFN is considered to be a major linkage point between innate and adaptive immunity, because IFN-α/β sustains B cell activation and differentiation [52], [53], expands antigen-specific CD8^+^ T cells [54], CD4^+^ T cells [55], and activation of NK cells [56]. Therefore, another intriguing finding of this study was the global alteration of immune responses in TLR4^−/−^ mice. This suggests that TLR4 molecule is largely dispensable for the efficient link between innate and adaptive immunity in JEV infection. Infection of TLR4^−/−^ mice with JEV exhibited the expansion of pDC and NK cells, and enhanced JEV-specific CD4^+^ and CD8^+^ T cell responses, which are involved in viral clearance at early and late phases of infection, respectively. Also, it is likely that increased number of pDCs contributed in part to the potent induction of type I IFN innate responses in TLR4^−/−^ mice. In addition, TLR4^−/−^ mice showed limited expansion of CD4^+^CD25^+^Foxp3^+^ Tregs, which have been known to suppress innate and effector T cells, thus preventing or controlling reactivity to self-antigen and pathogens, and thereby blunting severe inflammation and maintaining antigen-specific T cell homeostasis [40]. The role of CD4^+^CD25^+^Foxp3^+^ Tregs in acute viral diseases is still debatable [57], [58]. Recent work implicates CD4^+^Foxp3^+^ Tregs in the control of WNV pathogenesis, wherein peripheral expansion of Treg was associated with mild inflammation, but reduced Treg levels were associated with WNV encephalitis [57]. However, while CD4^+^Foxp3^+^ Tregs that were adoptively transferred 2 days prior to JEV infection made the recipients vulnerable to JE, CD4^+^Foxp3^+^ Tregs that were adoptively transferred 2 days after infection provided resistance to JE (unpublished personal data). This suggests that CD4^+^Foxp3^+^ Tregs elicit dual-phased roles during the progression of JEV-induced neurological disorders. More importantly, Treg induction during a viral infection is considered to be a detrimental response that promotes virus persistence without benefits to the host [59], [60]. One trivial explanation of CD4^+^Foxp3^+^ Treg role is that initially low number of CD4^+^Foxp3^+^ Tregs in TLR4^−/−^ mice may promote the expansion of effector CD4^+^ and CD8^+^ T cells specific for JEV antigen as well as innate immune responses, thereby inducing enhanced anti-viral response and virus-specific CTL to promote early viral clearance.

JE pathogenesis in the murine model may be altered by the route of peripheral administration, virus-propagation condition, and viral strains [7], [20], [21]. It is also possible that the genetic background of mice affects the immunopathogenesis of JE. However, we found that two backgrounds of mouse strains used for TLR3^−/−^ and TLR4^−/−^ mice showed comparable mortality and similar clinical signs after JEV infection, which indicates that JE pathogenesis is unaffected by genetic background of mouse strains used in this study. Although JEV infected *via* i.p. route does not directly reflect natural infection mediated by intradermal or intramuscular route after biting of mosquitoes, JEV infected *via* i.p. route displays entirely similar pathogenesis to natural infection, due to peripheral amplification in the spleen. Also, since mice infected i.p. with JEV usually exhibited neurological disorder at 4–5 days pi, rapid innate immune responses are more critical to control JE progression than adaptive T cell responses, which take time to develop. Indeed, the role of T cells in flavivirus encephalitis is less clear. This is, in part, due to variation of virus strain, the infection dose, the route of administration, mouse strain and age of the mice. Therefore, considering that the character of CD4^+^ and CD8^+^ T cells specific for JEV is also governed by innate immune responses initiated by recognition of PRRs, triggering of each PRR by direct viral components and/or host factors derived from infection could affect innate immune responses to shape adaptive immune responses, thereby influencing JE pathogenesis. A better understanding of the mechanisms that govern the induction of protective immunity plays a critical role in developing novel therapeutic strategies against JE.

## Materials and Methods

### Animals and ethics statement

C57BL/6 (H-2^b^) and BALB/c (H-2^d^) mice (4–6 weeks old) were purchased from Samtako (O-San, Korea). TLR2 (H-2^b^), TLR3 (H-2^d^ and H-2^b^), TLR4 (H-2^b^), TLR7 (H-2^d^), and TLR9 (H-2^b^)-deficient mice have been described elsewhere [33]. TLR3/4^−/−^ mice that are deficient in both TLR3 and TLR4 molecules were generated by backcrossing with TLR3 and TLR4-deficient mice. All mice were genotyped and bred in the animal facilities of Chonbuk National University. All experimental procedures were pre-approved and adhered to the guidelines set by the Institutional Animal Care and Use Committees (IACUC), Chonbuk National University (Permission code 2013-0028), which is fully accredited by the Korea Association for Laboratory Animal Sciences (KALAS).

### Cells and viruses

JEV Beijing-1 strain was obtained from Green Cross Research Institute (Suwon, Korea) and propagated in the mosquito cell line (C6/36) using DMEM supplemented with 2% FBS, penicillin (100 U/ml), and streptomycin (100 U/ml). The C6/36 cultures were infected with JEV Beijing-1 at a multiplicity of infection (MOI) of 0.1, and were incubated in a humidified CO_2_ incubator for 1 h at 28°C. After absorption, the inoculum was removed, and 7 ml of a maintenance medium containing 2% FBS was added. Approximately 6–7 days pi, cultures of the host cells showing an 80–90% cytopathic effect were harvested. The virus stocks were titrated by conventional plaque assay or focus-forming assay, and were stored in aliquots at −80°C until use.

### Antibodies and reagents

The mAbs used for the flow cytometric analysis and other experiments were obtained from eBioscience (San Diego, CA) or BD Biosciences (San Diego, CA) which include: fluorescein isothiocyanate (FITC) conjugate-anti-CD3ε (145-2C11), CD4 (RM4-5), CD8 (53-6.7), CD44 (IM7), CD62L (MEL-14), CD69 (H1.2F3), Ly-6G (1A8), anti-rabbit IgG, phycoerythrin (PE) conjugate-anti-mouse-CD11b (M1/70), Foxp3 (FJK-16s), IFN-γ (XMG1.2), goat anti-mouse IgG, peridinin chlorophyll protein complex (PerCP) conjugate-anti-Ly-6C (HK1.4), PE-Cyanine dye (Cy7)-anti-mouse NK1.1 (PK136), allophycocyanin (APC) conjugate-anti-mouse-CD25 (PC62.5), Ly-6G (Gr-1), TNF-α (MP6-XT22). The peptides of the defined I-A^b^-restricted epitopes JEV NS1_132–145_ (TFVVDGPETKECPD), H-2D^b^-restricted epitope JEV NS4B_215–223_ (SAVWNSTTA) [61], and H-2^d^-restricted epitope JEV E_60–68_ (CYHASVTDI) [62] were chemically synthesized at Peptron Inc. (Daejeon, Korea). Poly(I:C) was purchased from Sigma-Aldrich (St. Louis, MO). JEV-specific primers for the detection of viral RNA (JEV10,564–10,583 forward, 5′-CCC TCA GAA CCG TCT CGG AA-3′ and JEV10,862–10,886 reverse, 5′-CTA TTC CCA GGT GTC AAT ATG CTG T-3′) [27] and primers specific for cytokines, type I IFNs (IFN-α/β), and ISGs (**Table S2**) were synthesized at Bioneer Corp. (Daejeon, Korea) and used for PCR amplification of target genes.

### Quantitative real-time RT-PCR for viral burden and cytokine expression

Viral burden, cytokine (IL-1β, IL-6, TNF-α, IFN-α, and IFN-β) and chemokine (CCL2, CCL3, CCL4, CCL5, and CXCL10) expression in inflammatory and lymphoid tissues were determined by conducting quantitative SYBR Green-based real-time RT-PCR (real-time qRT-PCR). Mice were infected intraperitoneally (i.p.) with JEV (1.4 × 10^7^ PFU) and tissues including brain, spinal cord, and spleen were harvested at 2, 3, 4, and 6 days pi following extensive cardiac perfusion with Hanks balanced salt solution (HBSS). Total RNAs extracted from tissues using easyBLUE (iNtRON, INC., Daejeon, Korea) were employed for real-time qRT-PCR using a CFX96 Real-Time PCR Detection system (Bio-Rad Laboratories, Hercules, CA). Following reverse transcription of total RNAs with High-Capacity cDNA Reverse Transcription Kits (Applied Biosystems, Foster, CA), the reaction mixture contained 2 µl of template cDNA, 10 µl of 2× SYBR Primix Ex Taq, and 200 nM primers at a final volume of 20 µl. The reactions were denatured at 95°C for 30 s, and then subjected to 45 cycles of 95°C for 5 s, and 60°C for 20 s. After the reaction cycle was completed the temperature was increased from 65°C to 95°C at a rate of 0.2°C/15 s, and the fluorescence was measured every 5 s to construct a melting curve. A control sample that contained no template DNA was run with each assay, and all determinations were performed at least in duplicate to ensure reproducibility. The authenticity of the amplified product was determined by melting curve analysis. The relative ratio of viral RNA in the infected samples to uninfected samples was determined. All data were analyzed using the Bio-Rad CFX Manager, version 2.1 analysis software (Bio-Rad Laboratories).

### Cytokine and type I IFN ELISA

#### (i) IL-6 cytokine ELISA

Sandwich ELISA was used to determine the levels of IL-6 cytokines in sera and culture supernatants. ELISA plates were coated with IL-6 (MP5-20F3) anti-mouse Ab purchased from eBioscience, and then incubated overnight at 4°C. The plates were washed three times with PBS containing 0.05% Tween (PBST), after which they were blocked with 3% nonfat-dried milk for 2 h at 37°C. The sera and culture supernatant and standards for recombinant cytokine proteins (PeproTech, Rocky Hill, NJ) were added to the plates, which were then incubated for 2 h at 37°C. The plates were washed with PBST again and biotinylated IL-6 (MP5-32C11) Ab was added. Next, the plates were incubated overnight at 4°C, followed by washing with PBST and subsequent incubation with peroxidase-conjugated streptavidine (eBioscience) for 1 h. Color development was then performed by the addition of a substrate (ABTS) solution. Cytokine concentrations were determined with an automated ELISA reader and SoftMax Pro3.4 according to comparisons with two concentrations of standard cytokine proteins.

#### (ii) Type I IFN (IFN-β) ELISA

A commercial ELISA kit (PBL Biomedical Laboratories, Piscataway, NJ) was used to measure levels of secreted IFN-β protein in sera and cell culture supernatants, according to the manufacturer's protocol.

### Analysis of leukocytes infiltrated into the CNS

Mice infected with JEV were perfused with 30 ml of HBSS on day 3 pi *via* cardiac puncture of the left ventricle. Brains were then harvested, and homogenized by gently pressing them through 100-mesh tissue sieve, after which they were digested with 25 µg/ml of collagenase type IV (Worthington Biochem, Freehold, NJ), 0.1 µg/ml trypsin inhibitor *Nα*-*p*-tosyl-L-lysine chloromethyl ketone, 10 µg/ml DNase I (Amresco, Solon, OH), and 10 mM HEPE in HBSS for 1 h at 37°C with shaking. Cells were separated by using Optiprep density gradient (18/10/5%) centrifugation at 400 ×g for 30 min (Axis-Shield, Oslo, Norway), after which cells were collected from 18% to 10% interface and washed twice with PBS. Cells were then counted and stained for CD11b, Gr-1, Ly6G, Ly6C, CD3, CD4, CD8, and NK1.1 with directly conjugated antibodies (eBioscience) for 30 min at 4°C. Finally, the cells were fixed with 10% formaldehyde. Data collection and analysis were performed with a FACSCalibur flow cytometer (Becton Dickson Medical Systems, Sharon, MA) and FlowJo (Tree Star, San Carlos, CA) software.

### Histological examinations and confocal microscopy

Brain derived from mock and JEV-infected mice were embedded in paraffin and 10-µm sections were prepared and stained with hematoxylin and eosin (H&E). Sections were analyzed using a Nikon Eclipse E600 microscope (Nikon, Tokyo, Japan). For confocal microscopy of brain tissue, brains were collected and frozen in optimum cutting temperature (OCT) compound (Sakura Finetechnical Co., Tokyo, Japan) following vigorous perfusion with HBSS. 6–7 µm thick sections were then cut, air-dried, and fixed in cold solution (1∶1 mixture of acetone and methanol) for 15 min at −20°C. Non-specific binding was blocked with 10% normal goat serum, and samples were then permeabilized with 0.1% Triton X-100. Staining was performed by incubating sections overnight in moist chamber at 4°C with anti-JEV and biotin conjugated anti-mouse CD11b (BD Biosciences, San Diego, CA) antibody. Primary antibodies were detected with secondary FITC-conjugated streptavidin and PE-conjugated goat anti-mouse Ab. Nuclei were counterstained with DAPI (4′6-diamidino-2-phenylindole) (Sigma-Aldrich). Finally, the fluorescence was observed by confocal laser scanning microscope (CalZeiss, Zena, Germany).

### Evaluation of BBB permeability

Blood–brain barrier (BBB) permeability was determined by visualizing and quantifying extravasated Evans blue dye into the brain, as described earlier with some modification [20], [21]. Briefly, JEV-infected mice were injected i.p. with 800 µl of 1% (w/v) Evans Blue dye (Sigma-Aldrich) 2 and 3 days pi, and perfused *via* intracardiac puncture with HBSS 1 h later. Brains were subsequently removed, weighed, and stored at −80°C following visualization with a high resolution digital camera. For Evans blue quantification, brain tissues were homogenized in 1 ml of PBS, and 1 ml of 100% trichloroacetic acid (TCA) (Sigma-Aldrich) was then added to the homogenate to precipitate proteins. The mixture was then vigorously shaken to precipitate the proteins for 2 min and cooled for 30 min at 4°C. After centrifugation (30 min at 4,000 ×g), the absorbance of the supernatant was measured at 620 nm using a spectrophotometer. The content of Evans blue was valued as micrograms of dye per gram of brain tissue by using a standard curve.

### Primary cell culture and infection

#### (i) Myeloid-derived DCs and macrophages

Myeloid-derived DCs (BMDC) and macrophages (BMDM) were prepared from bone marrow cells of TLR3^−/−^, TLR4^−/−^, and WT mice, as described earlier with some modification [27]. Briefly, for BMDC, bone marrow cells (3×10^5^ cells/ml) from femurs and tibiae were cultured in RPMI 1640 supplemented with 2 ng/ml GM-CSF and 10 ng/ml IL-4. On day 3, another 6 ml of fresh complete medium containing 2 ng/ml GM-CSF and 10 ng/ml IL-4 was added, and half of the medium was changed on day 6. On day 8, non-adherent and loosely adherent DCs were harvested by vigorous pipetting. Cells were then characterized by flow cytometric analysis, which revealed that the culture generally consisted of >85% CD11c^+^ cells (25% CD11c^+^CD11b^+^ and 65% CD11c^+^CD8α^+^). BMDM was prepared by culturing bone marrow cells in DMEM supplemented containing 30% L929 cell-conditioned medium (LCCM) as a source of macrophage-colony stimulating factor (M-CSF). On day 3, another 6 ml of fresh complete medium containing 30% LCCM was added, and half of the medium was changed on day 6. The cultured cells were harvested following 8-day incubation and were analyzed by flow cytometry. The prepared BMDMs were composed of >85% F4/80^+^ cells that consisted of 99.2% F4/80^+^CD11b^+^ and ∼1% F4/80^+^CD11c^+^ cells. Prepared BMDC and BMDM were infected with JEV at a MOI of 1.0 for viral replication and 10 MOI for cytokine expression.

#### (ii) Primary cortical neurons

Primary cortical neurons were prepared from 15-day-old embryo as described previously [29]. Cortical neurons were seeded in 12-well poly-D-lysine/laminin-coated plates in DMEM containing 5% FBS and 5% horse serum for 24 h, and then cultured for 4 days with Neurobasal medium containing B27 supplement and L-glutamine (Invitrogen, Carlsbad, CA). Primary cortical neurons were infected with JEV at a 0.1 MOI for viral replication and type I IFN responses.

### Western blotting

BMDC and BMDM infected with JEV were lysed in RIPA buffer (10 mM Tris, 150 mM NaCl, 0.02% sodium azide, 1% sodium deoxycholate, 1% Triton X-100, 0.1% SDS, pH 7.4) supplemented with protease inhibitors (iNtRON Biotech, Daejeon, Korea). Samples (15 µg) were resolved by electrophoresis on 10 to 12.5% SDS-polyacrylamide gels. After proteins were transferred to PVDF Immobilon-P Transfer Membrane (Millipore, Billerica, MA), blots were blocked with 5% non-fat dried milk or 3% BSA overnight at 4°C, and probed with the following panel of primary antibodies: rabbit anti-IRF-3, phospho-IRF-3 (Ser396), STAT1, phospho-STAT1 (Tyr701), mouse anti-IκBα (Amino-terminal Antigen) antibodies (Cell Signaling, Danvers, MA), and rabbit anti-IRF-7, phospho-IRF-7 (Ser471+Ser472) antibodies (Bioss Inc, Woburn, MA). Western blots were incubated with peroxidase-conjugated secondary antibodies (SouthernBiotech, Birmingham, AL) and visualized with WEST-ZOL Plus Immunoblotting detection reagents (iNtRON Biotech) using chemi-documentation system (Fusion Fx7, Vilber Lourmat, Cedex1, France).

### JEV-specific antibody and CD4^+^/CD8^+^ T-cell responses

JEV-specific IgM and IgG levels in sera of infected mice were determined by conventional ELISA using JEV E glycoprotein antigen (Abcam, Cambridge, UK). JEV-specific CD4+ and CD8+ T cell responses were determined by intracellular IFN-γ and TNF-α staining in response to antigen stimulation. Mice were infected i.p. with 2.8 ×10^6^ PFU of JEV and were sacrificed at day 7 pi and splenocytes were prepared. The erythrocytes were depleted by treating single-cell suspensions with ammonium chloride-containing Tris buffer (NH_4_Cl-Tris) for 5 min at 37°C. The splenocytes were cultured in 96-well culture plates (5×10^5^ cells/well) in the presence of synthetic peptide epitopes (NS1_132–145_ and NS4B_215–225_ or E_60–68_) for 12 h and 6 h in order to observe CD4^+^ and CD8^+^ T cell responses, respectively. CD4^+^ responses of BALB/c genetic background (H-2^d^) strain groups were evaluated by stimulation with UV-irradiated virus for 12 h at 37°C. Monensin at the concentration of 2 µM was added to antigen-stimulated cells 6 h before harvest. The cells were washed twice with PBS, and surface stained for FITC-anti-CD4 or CD8 antibodies for 30 min at 4°C, and then washed twice with PBS containing monensin. After fixation, the cells were washed twice with permeabilization buffer (eBioscience) and then stained with PE-anti-IFN-γ, or APC-anti-TNF-α in permeabilization buffer for 30 min at room temperature. Finally, the cells were washed twice with PBS and fixed using fixation buffer. Sample analysis was performed with FACS Calibur flow cytometer (Becton Dickson Medical Systems, Sharon, MA) and FlowJo (Tree Star, San Carlos, CA) software.

### Analysis of myeloid-derived and immune cell subsets in lymphoid tissues

Splenocytes from infected mice were isolated and digested with collagenase (Roche) and type I DNase in serum-free RPMI media at 37°C for 40 min with mechanical disruption. Cells were washed twice with RPMI media containing 10% FBS before FACS staining. Myeloid-derived and immune cells were stained antibodies specific for CD11c, CD11b, B220, CD3, CD4, CD8α, NK1.1, DX5, Gr-1, Ly-6C, PDCA-1, CD25, and intracellular Foxp3. Finally, the cells were fixed with 10% formaldehyde, and analyzed with FACS Calibur flow cytometer and FlowJo software.

### Statistical analysis

All data were expressed as the average ± standard deviation, and statistically significant differences between groups were analyzed by unpaired two-tailed Student's *t*-test for *in vitro* experiments and immune cell analysis or ANOVA and post-hoc test for multiple comparisons of the mean. The statistical significance of viral burden and *in vivo* cytokine gene expression were evaluated by Mann-Whitney test or unpaired two-tailed Student's t-test. Kaplan-Meier survival curves were analyzed by the log-rank test. A *p*-value ≤0.05 was considered significant. All data were analyzed using Prism software (GraphPadPrism4, San Diego, CA).

## Supporting Information

Figure S1 Susceptibility of TLR2^−/−^, TLR3^−/−^, TLR4^−/−^, TLR7^−/−^, TLR9^−/−^ mice to lethal encephalitis caused by JEV infection. TLR2^−/−^ (A), TLR3^−/−^ (B), TLR4^−/−^ (C), TLR7^−/−^ (D), and TLR9^−/−^ (E) mice (*n* = 7–13) were infected with JEV (1.4×10^7^ pfu) and were then monitored for mortality over 15 days. Data represent the proportion of surviving mice relative to challenged mice. Survival differences were statistically significant (B, *p = *0.0153; C, *p* = 0.0819).
(PDF)

Figure S2 Susceptibility and viral burden of TLR3^−/−^ mice (C57BL/6 genetic background) in lethal encephalitis caused by JEV infection. TLR3^−/−^ mice derived from C57BL/6 genetic background (H-2^b^) were infected with two doses, 1.4×10^7^ pfu (A) and 2.8×10^7^ pfu (B), of JEV, and then monitored for mortality, paralysis rate and body weight. The survival rate was examined over 15 days, and ratio of mice showing neurological disease during JE progression was examined every 6 h from 4 to 7 days pi. The change of body weight was expressed as the average percentage ± SD of weight relative to the time of challenge (*n* = 9–13). (C) Viral burden in lymphoid and inflammatory tissues during JE progression. Viral burden in spleen, brain, and spinal cord of TLR3^−/−^ (C57BL/6 genetic background) mice infected with JEV was assessed by real-time qRT-PCR at the indicated days pi. The viral RNA load was expressed by viral RNA copy number per microgram of total RNA (*n* = 5). Each symbol represents the level of an individual mouse; horizontal line indicates the median of each group.
(PDF)

Figure S3 TLR4 is dispensable to induce adequate adaptive immune responses specific for JEV antigen. (A) JEV-specific IgM and IgG levels. The levels of JEV-specific IgM and IgG in sera of TLR3^−/−^ and TLR4^−/−^ mice infected with sub-lethal dose of JEV (2.8×10^6^ pfu) were determined by ELISA at the indicated days pi. (B and C) JEV antigen-specific CD4^+^ T cell responses. Splenocytes were stimulated with UV-inactivated JEV (5 moi) for TLR3^−/−^ mice and NS1_132–145_ peptide (2 µg/ml) for TLR4^−/−^ mice at 7 days pi. The frequency (B) and total number (C) of JEV-specific CD4^+^ T cells were enumerated by the combined staining of surface CD4 and intracellular cytokines (IFN-γ and TNF-α). (D and E) JEV antigen-specific CD8^+^ T cell responses. Splenocytes were stimulated with E_60–68_ and NS4B_215–225_ peptides for TLR3^−/−^ and TLR4^−/−^ mice at 7 days pi, respectively. The frequency (D) and total number (E) of JEV-specific CD8^+^ T cells were enumerated by the combined staining of surface CD8 and intracellular cytokines (IFN-γ and TNF-α). The values in representative dot-plots denote the average of the indicated cell populations obtained from three individual experiment (*n* = 3–4). The bar in the graph represents the average ± SD of total number of the indicated cell population. *, *p*<0.001; **, *p*<0.01; ***, *p*<0.05 compared with the levels of the indicated group.
(PDF)

Table S1 Summary of the percentage and activation of splenic lymphocyte subsets in TLR3 and TLR4-deficient mice following JEV infection. TLR3 and TLR4-deficient (KO) mice (*n* = 3) were infected i.p. with JEV (1.4×10^7^ pfu/mouse). Splenocytes were prepared 3 days pi, stained with the indicated Abs, and were then analyzed by flow cytometry. Results are expressed as the average ± SD of positive cells (CD69^+^, CD62L^Low^, CD44^high^, or CD80^+^) in a given cell subset (CD4^+^, CD8^+^, or CD19^+^).
(PDF)

Table S2 Specific primers for cytokines, chemokines, and type I IFNs, and ISGs used in real-time qRT-PCR.
(PDF)
