# Supplementary figures and images for: Distinct Dictation of Japanese Encephalitis Virus-Induced Neuroinflammation and Lethality via Triggering TLR3 and TLR4 Signal Pathways
Source: PLoS Pathog. 2014 Sep 4;10(9):e1004319. doi: 10.1371/journal.ppat.1004319 (PMC4154777; doi:10.1371/journal.ppat.1004319)

**A**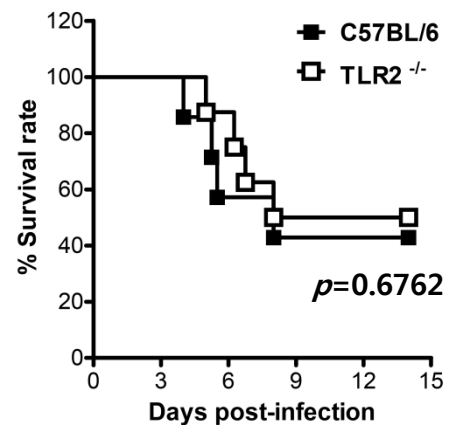**B**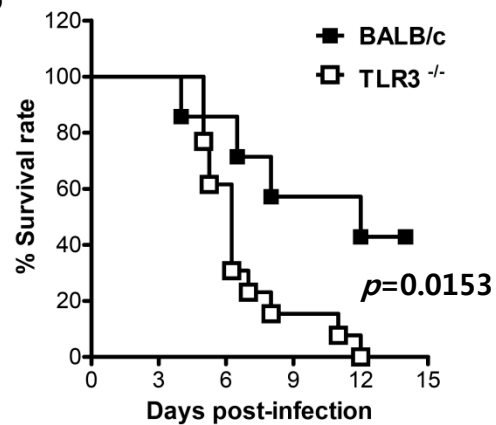**C**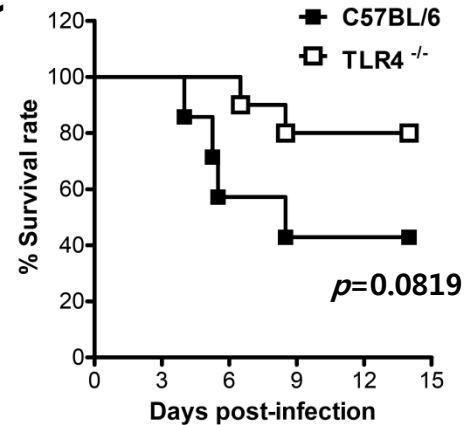**D**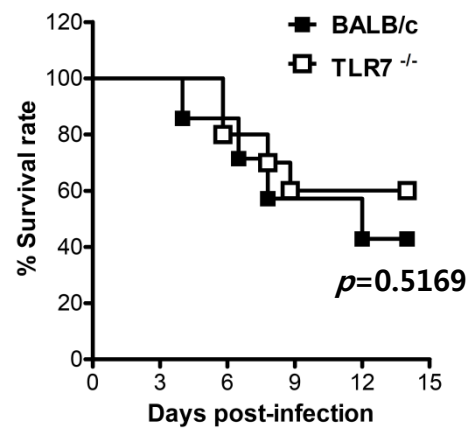**E**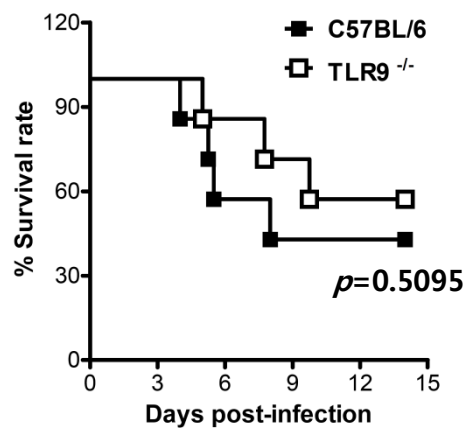

Supplement: Figure S1 — Susceptibility of TLR2−/−, TLR3−/−, TLR4−/−, TLR7−/−, TLR9−/− mice to lethal encephalitis caused by JEV infection. TLR2−/− (A), TLR3−/− (B), TLR4−/− (C), TLR7−/− (D), and TLR9−/− (E) mice (n = 7–13) were infected with JEV (1.4×10^7^ pfu) and were then monitored for mortality over 15 days. Data represent the proportion of surviving mice relative to challenged mice. Survival differences were statistically significant (B, p = 0.0153; C, p = 0.0819). (PDF) [file ppat.1004319.s001.pdf]

**A**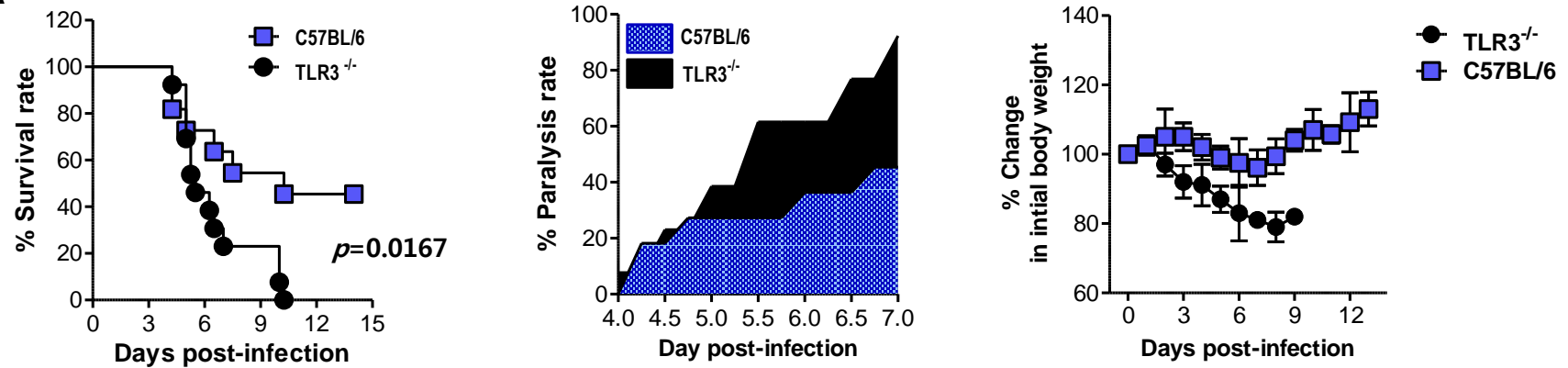**B**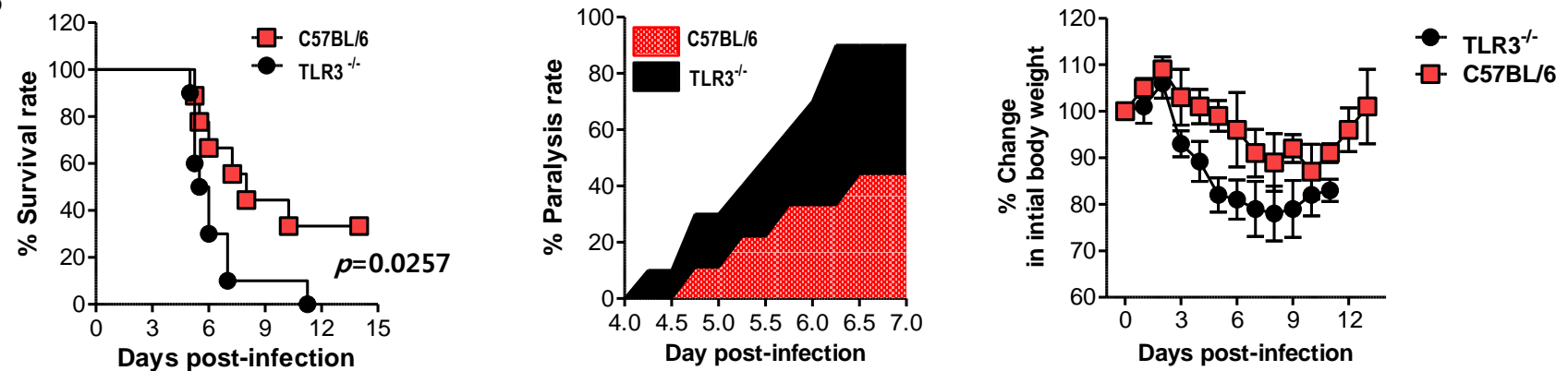**C**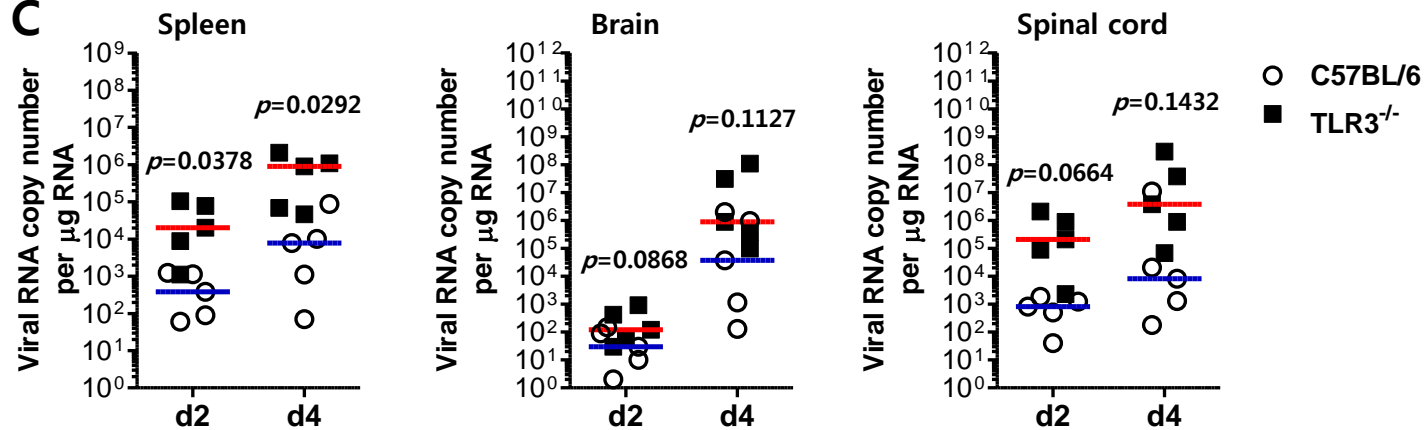

Supplement: Figure S2 — Susceptibility and viral burden of TLR3−/− mice (C57BL/6 genetic background) in lethal encephalitis caused by JEV infection. TLR3−/− mice derived from C57BL/6 genetic background (H-2b) were infected with two doses, 1.4×10^7^ pfu (A) and 2.8×10^7^ pfu (B), of JEV, and then monitored for mortality, paralysis rate and body weight. The survival rate was examined over 15 days, and ratio of mice showing neurological disease during JE progression was examined every 6 h from 4 to 7 days pi. The change of body weight was expressed as the average percentage ± SD of weight relative to the time of challenge (n = 9–13). (C) Viral burden in lymphoid and inflammatory tissues during JE progression. Viral burden in spleen, brain, and spinal cord of TLR3−/− (C57BL/6 genetic background) mice infected with JEV was assessed by real-time qRT-PCR at the indicated days pi. The viral RNA load was expressed by viral RNA copy number per microgram of total RNA (n = 5). Each symbol represents the level of an individual mouse; horizontal line indicates the median of each group. (PDF) [file ppat.1004319.s002.pdf]

**A**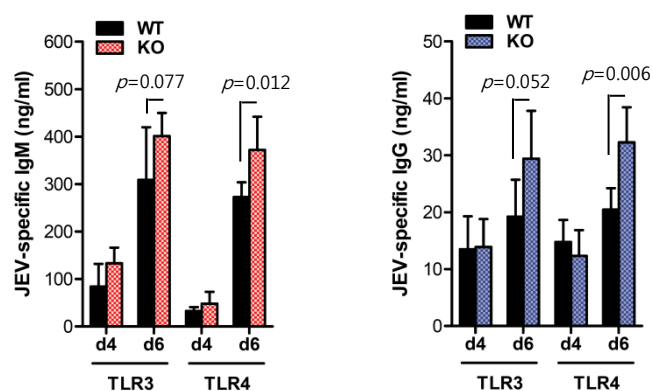**B**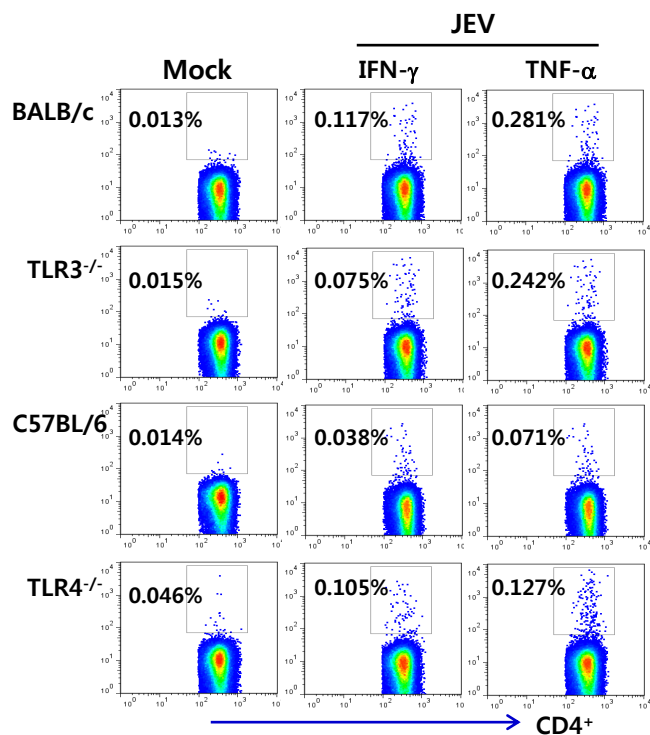**C**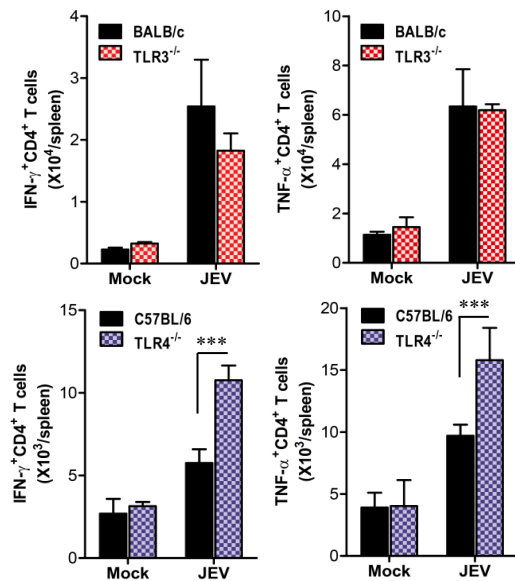**D**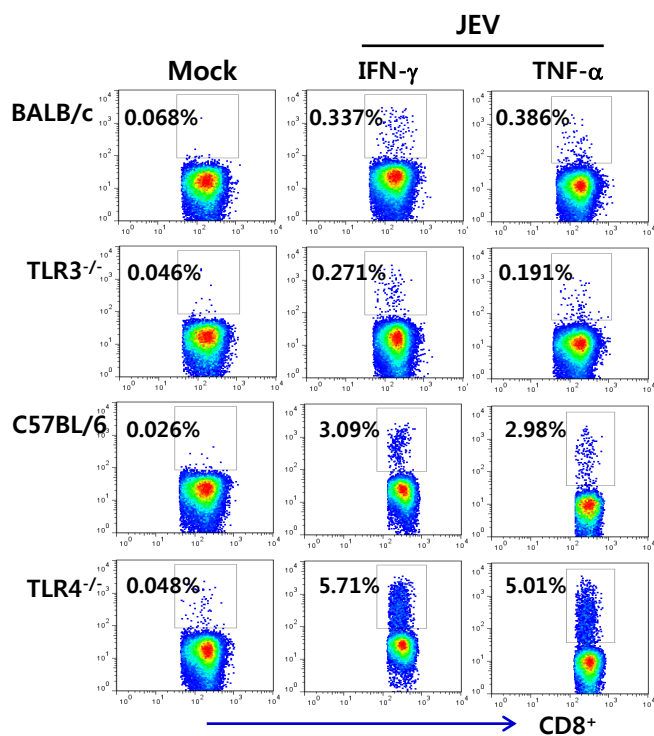**E**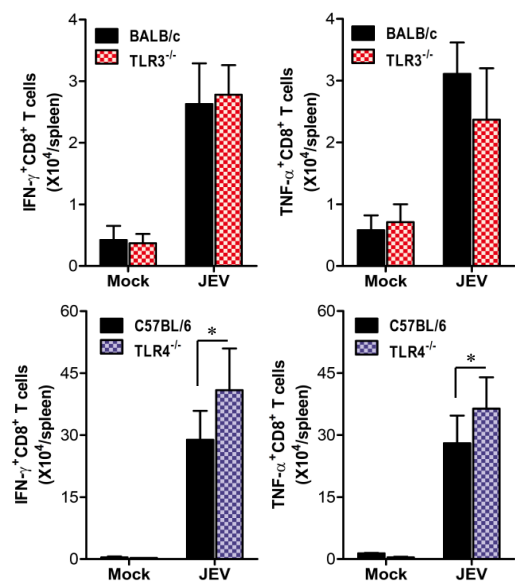

Supplement: Figure S3 — TLR4 is dispensable to induce adequate adaptive immune responses specific for JEV antigen. (A) JEV-specific IgM and IgG levels. The levels of JEV-specific IgM and IgG in sera of TLR3−/− and TLR4−/− mice infected with sub-lethal dose of JEV (2.8×10^6^ pfu) were determined by ELISA at the indicated days pi. (B and C) JEV antigen-specific CD4+ T cell responses. Splenocytes were stimulated with UV-inactivated JEV (5 moi) for TLR3−/− mice and NS1132–145 peptide (2 µg/ml) for TLR4−/− mice at 7 days pi. The frequency (B) and total number (C) of JEV-specific CD4+ T cells were enumerated by the combined staining of surface CD4 and intracellular cytokines (IFN-γ and TNF-α). (D and E) JEV antigen-specific CD8+ T cell responses. Splenocytes were stimulated with E60–68 and NS4B215–225 peptides for TLR3−/− and TLR4−/− mice at 7 days pi, respectively. The frequency (D) and total number (E) of JEV-specific CD8+ T cells were enumerated by the combined staining of surface CD8 and intracellular cytokines (IFN-γ and TNF-α). The values in representative dot-plots denote the average of the indicated cell populations obtained from three individual experiment (n = 3–4). The bar in the graph represents the average ± SD of total number of the indicated cell population. *, p<0.001; **, p<0.01; ***, p<0.05 compared with the levels of the indicated group. (PDF) [file ppat.1004319.s003.pdf]
