# Supplementary material for: Distinct Dictation of Japanese Encephalitis Virus-Induced Neuroinflammation and Lethality via Triggering TLR3 and TLR4 Signal Pathways
Source: PLoS Pathog. 2014 Sep 4;10(9):e1004319. doi: 10.1371/journal.ppat.1004319 (PMC4154777; doi:10.1371/journal.ppat.1004319)
Supplement: Table S1 — Summary of the percentage and activation of splenic lymphocyte subsets in TLR3 and TLR4-deficient mice following JEV infection. TLR3 and TLR4-deficient (KO) mice (n = 3) were infected i.p. with JEV (1.4×10^7^ pfu/mouse). Splenocytes were prepared 3 days pi, stained with the indicated Abs, and were then analyzed by flow cytometry. Results are expressed as the average ± SD of positive cells (CD69+, CD62LLow, CD44high, or CD80+) in a given cell subset (CD4+, CD8+, or CD19+). (PDF) [file ppat.1004319.s004.pdf]

Supplementary Table 1. Summary of the percentage and activation of splenic lymphocyte subsets in TLR3 and TLR4-deficient mice following JEV infection

% Positive cells	TLR3				TLR4			
	WT		KO		WT		KO	
	Mock	JEV	Mock	JEV	Mock	JEV	Mock	JEV
CD4$^+$	11.97±1 89	11.12±1.71	22.96±3.69	13.84±2.45*	14.15±0.74	10.52±0.26**	18.31±1.58	14.62±0.65*
CD4$^+$CD69$^+$	4.04±0.19	8.30±1.99*	4.03±0.48	8.93±0.89**	6.40±1.59	10.21±1.78	5.63±0.32	4.71±0.53
CD4$^+$CD62L^{low}	25.42±3.39	24.14±0.87	19.98±0.30	29.09±1.06***	25.92±4.87	38.88±1.00*	36.54±3.59	35.68±3.24
CD4$^+$CD44^{high}	12.51±0.27	13.52±0.51*	11.93±0.99	13.36±1.29	10.77±1.53	10.97±2.20	10.97±1.85	9.21±1.58
CD8$^+$	5.50±0.72	6.59±1.36	9.09±1.17	7.65±1.11	7.64±0.44	6.16±1.04	9.25±1.01	8.82±0.54
CD8$^+$CD69$^+$	1.42±0.19	3.49±1.21*	5.59±0.66	6.42±2.30	2.26±0.40	4.50±1.30*	1.22±0.06	2.57±0.35**
CD8$^+$CD62L^{low}	31.07±2.36	26.64±2.30	23.64±2.55	24.57±1.08	13.13±2.79	23.37±0.97**	19.65±2.99	16.67±3.79
CD8$^+$CD44^{high}	21.45±1.87	19.16±1.99	19.88±0.89	21.39±2.44	9.55±0.73	9.23±0.40	21.38±2.15	14.87±0.82**
CD19$^+$	44.01±5.13	42.13±5.17	42.18±0.99	43.78±2.82	53.09±1.80	61.23±6.69	48.64±0.30	48.55±3.94
CD19$^+$CD69$^+$	0.15±0.01	0.56±0.25*	0.09±0.01	0.20±0.05*	0.33±0.08	1.19±0.65	0.27±0.16	0.43±0.14
CD19$^+$CD80$^+$	0.63±0.14	1.06±0.08**	1.88±0.48	2.34±1.00	1.40±0.29	1.46±0.08	0.75±0.20	0.58±0.12

[a]TLR3 and TLR4-deficient (KO) mice (*n*=3) were infected i.p. with JEV (1.4×10^7 pfu/mouse). Splenocytes were prepared 3 days p.i., stained with indicated Abs, and analyzed by flow cytometry. Results are expressed as the average ± SD of positive cells (CD69$^+$, CD62L^{Low}, CD44^{high}, or CD80$^+$) in a given cell subset (CD4$^+$, CD8$^+$, or CD19$^+$)

*$p<0.05$; **$p<0.01$; ***$p<0.001$; Student's *t*-test, calculated between mock-infected and the indicated group.
