# Supplementary material for: Distinct Dictation of Japanese Encephalitis Virus-Induced Neuroinflammation and Lethality via Triggering TLR3 and TLR4 Signal Pathways
Source: PLoS Pathog. 2014 Sep 4;10(9):e1004319. doi: 10.1371/journal.ppat.1004319 (PMC4154777; doi:10.1371/journal.ppat.1004319)
Supplement: Table S2 — Specific primers for cytokines, chemokines, and type I IFNs, and ISGs used in real-time qRT-PCR. (PDF) [file ppat.1004319.s005.pdf]

**Supplementary Table S2.** Specific primers for cytokines, chemokines, type I IFNs, and ISGs used in real-time qRT-PCR.

| Gene name[a] | Primer sequence (5'-3')[b] | Position cDNA | Gene Bank ID |
|---|---|---|---|
| IL-1β | FP: TCC AAG AAA GGA CGA ACA TTC G | 535-559 | NM_008361 |
| | RP: TGA GGA CAT CTC CCA CGT CAA | 679-700 | |
| IL-6 | FP: TGG GAA ATC GTG GAA ATG AG | 209-228 | NM_031168 |
| | RP: CTC TGA AGG ACT CTG GCT TTG | 442-462 | |
| TNF-α | FP: CGT CGT AGC AAA CCA CCA AG | 438-457 | NM_013693 |
| | RP: TTG AAG AGA ACC TGG GAG TAG ACA | 564-587 | |
| IFN-α | FP: TGTCTGATGCAGCAGGTGG | 367-385 | NM_008334.3 |
| | RP: AAGACAGGGCTCTCCAGAC | 514-532 | |
| IFN-β | FP: TCCAAGAAAGGACGAACATTCG | 106-121 | NM_010510 |
| | RP: TGAGGACATCTCCCACGTCAA | 399-419 | |
| CCL2 | FP: AAA AAC CTG GAT CGG AAC CAA | 347-367 | NM_011333 |
| | RP: CGG GTC AAC TTC ACA TTC AAA G | 426-447 | |
| CCL3 | FP: CCA AGT CTT CTC AGC GCC AT | 158-177 | NM_011337.2 |
| | RP: GAA TCT TCC GGC TGT AGG AGA AG | 206-228 | |
| CCL4 | FP: TTC TGT GCT CCA GGG TTC TC | 128-147 | NM_013652.2 |
| | RP: GAG GAG GCC TCT CCT GAA GT | 388-407 | |
| CCL5 | FP: CCC TCA CCA TCA TCC TCA CT | 77-96 | NM_013653.3 |
| | RP: CTT CTT CTC TGG GTT GGC AC | 275-294 | |
| CXCL10 | RP: CAT TCT TTT TCA TCG TGG CA | 264-283 | NM_021274.2 |
| | FP: AAG TGC TGC CGT CAT TTT CT | 84-103 | |
| IRF3 | FP: GAT GGA GAG GTC CAC AAG GA | 1170-1189 | NM_016849 |
| | RP: GAG TGT AGC GTG GGG AGT GT | 1259-1278 | |
| IRF5 | FP: GGA AGA AAT GAA GCC AGC AG | 1931-1950 | NM_001252382.1 |
| | RP: ACC CTG GGG TAA TTG GAC TC | 2001-2020 | |
| IRF7 | FP: CCT CTT GCT TCA GGT TCT GC | 980-999 | NM_016850.3 |
| | RP:GCT GCA TAG GGT TCC TCG TA | 1080-1099 | |
| RIG-I | FP: CCA CCT ACA TCC TCA GCT ACA TGA | 194-217 | NM_172689 |
| | RP:TGG GCC CTT GTT GTT CTT CT | 260-279 | |
| MAD5 | FP: GGC ACC ATG GGA AGT GAT T | 1178-1196 | NM_027835 |
| | RP:ATT TGG TAA GGC CTG AGC TG | 1247-1266 | |
| PKR | FP: AAC TTC TTC ACA CGT GCT TC | 1517-1536 | NM_011163 |
| | RP: CAT TCA GCC AAG GTC TTC AG | 1678-1697 | |
| STAT1 | FP: AAG CGA ACT GGA TAC ATC A | 2093-2111 | U06924.1 |

| | | | |
|---|---|---|---|
| | RP: CCG GGA CAT CTC ATC AAA C | 2194-2212 | |
| 2’5’-Oas1 | FP: CCA TCC TCA AGT GGA CAA GAA CTG | 1235-1258 | AF466822.1 |
| | RP: TTG GGC TTT GGG CAC CTT C | 1359-1377 | |
| Oasl-1 | FP: CCA GGA AGA AGC CAA GCA CCA TC | 377-399 | NM_145209.3 |
| | RP: AGG TTA CTG AGC CCA AGG TCC ATC | 449-472 | |
| Mx1 | FP: CAG CAC CTG ATG GCC TAT CA | 2276-2295 | NM_010846.1 |
| | RP: ACG TCT GGA GCA TGA AGA ACT G | 2363-2342 | |
| Mx2 | FP: AGG CTC ACA ACC GCA TCT | 1858-1875 | NM_013606.1 |
| | RP:GCT CAG CAA ACA TTT TCA GG | 1910-1929 | |
| ISG49 | FP: GCC GTT ACA GGG AAA TAC TGG | 919-939 | NM_010501.2 |
| | RP:CCT CAA CAT CGG GGC TCT | 1126-1143 | |
| ISG54 | FP: GGG AAA GCA GAG GAA ATC AA | 1918-1937 | NM_008332.3 |
| | RP:TGA AAG TTG CCA TAC AGA AG | 2005-2024 | |
| ISG56 | FP: CAG AAG CAC ACA TTG AAG AA | 774-793 | NM_008331.3 |
| | RP:TGT AAG TAG CCA GAG GAA GG | 911-930 | |
| β-actin | FP: TGG AAT CCC TGT GGG ACC ATG AAA C | 885-909 | NM_007393.3 |
| | RP: TAA AAC GCA GCT CAG TAA CAG TCC G | 1209-1233 | |

[a] IL, interleukin; TNF-α, tumor necrosis factor-α; IFN, interferon

[b] FP, forward primer; RP, reverse primer
